# The Human Induced Pluripotent Stem Cell Test as an Alternative Method for Embryotoxicity Testing

**DOI:** 10.3390/ijms23063295

**Published:** 2022-03-18

**Authors:** Saskia Galanjuk, Etta Zühr, Arif Dönmez, Deniz Bartsch, Leo Kurian, Julia Tigges, Ellen Fritsche

**Affiliations:** 1 IUF—Leibniz Research Institute for Environmental Medicine, 40225 Duesseldorf, Germany; saskia.galanjuk@iuf-duesseldorf.de (S.G.); etta.zuehr@iuf-duesseldorf.de (E.Z.); arif.doenmez@iuf-duesseldorf.de (A.D.); julia.tigges@iuf-duesseldorf.de (J.T.); 2 Cologne Excellence Cluster on Cellular Stress Responses in Aging-Associated Diseases, Faculty of Medicine, University of Cologne, 50923 Cologne, Germany; deniz.bartsch@uk-koeln.de (D.B.); leo.kurian@uk-koeln.de (L.K.); 3 Center for Molecular Medicine (CMMC) and Institute for Neurophysiology, Faculty of Medicine, University of Cologne, 50923 Cologne, Germany; 4 Laboratory for Developmental and Regenerative RNA Biology, Center for Molecular Medicine Cologne, University of Cologne, 50923 Cologne, Germany; 5 Medical Faculty, Heinrich-Heine-University, 40225 Duesseldorf, Germany

**Keywords:** cardiomyocytes, hiPS Test, in vitro, hiPSC, video analyses, CardioVision, embryotoxicity, developmental toxicity

## Abstract

The evaluation of substances for their potency to induce embryotoxicity is controlled by safety regulations. Test guidelines for reproductive and developmental toxicity rely mainly on animal studies, which make up the majority of animal usage in regulatory toxicology. Therefore, there is an urgent need for alternative in vitro methods to follow the 3R principles. To improve human safety, cell models based on human cells are of great interest to overcome species differences. Here, human induced pluripotent stem cells (hiPSCs) are an ideal cell source as they largely recapitulate embryonic stem cells without bearing ethical concerns and they are able to differentiate into most cell types of the human body. Here, we set up and characterized a fetal bovine serum (FBS)-free hiPSC-based in vitro test method, called the human induced pluripotent stem cell test (hiPS Test), to evaluate the embryotoxic potential of substances. After 10 days in culture, hiPSCs develop into beating cardiomyocytes. As terminal endpoint evaluations, cell viability, qPCR analyses as well as beating frequency and area of beating cardiomyocytes by video analyses are measured. The embryotoxic positive and non-embryotoxic negative controls, 5-Fluorouracil (5-FU) and Penicillin G (PenG), respectively, were correctly assessed in the hiPS Test. More compounds need to be screened in the future for defining the assay’s applicability domain, which will inform us of the suitability of the hiPS Test for detecting adverse effects of substances on embryonic development.

## 1. Introduction

Embryotoxicity is defined as the adverse effect of substances on embryonic development [1]. Assessing compounds’ hazards and risks for embryotoxicity is of great scientific and socio-political interest, as they can cause birth defects or embryonic loss [2,3,4]. Of all births worldwide, 6% are concomitant with birth defects. About one-third of those are heart defects, making them the most common congenital anomalies. The etiologies of most congenital heart defects (70%) are idiopathic, with chromosomal or single-gene disorders contributing to the most known causes [2]. However, environmental exposure towards, e.g., chemicals or drugs during pregnancy has been gaining recognition as a contributing factor to adverse prenatal developmental outcomes [2,5].

The goal to protect human health and the environment in Europe is implemented in the European legislation [6]. As a consequence, the regulation ‘Registration, Evaluation, Authorization and Restriction of Chemicals’ (REACH) entered into force in 2007 in the European Union, stipulating the registration and approval of industrial, agricultural, and consumer products before entering the market by the European Chemicals Agency (ECHA) and the European Food Safety Authority (EFSA) [7]. Current testing guidelines are mainly based on in vivo studies using animals [8,9]. In vivo testing for reproductive and developmental toxicity [10] especially is time-consuming, resource intensive, and also raises ethical concerns due to the vast amount of animal usage [11]. Therefore, there is an urgent need for alternative in vitro test methods focusing on reproductive and developmental toxicity evaluation according to the 3R principles (replace, reduce, refine) [12]. This approach is supported by REACH to ultimately reduce or replace animal testing [7].

In the past, the ‘European Union Reference Laboratory for Alternatives to Animal Testing’ (EURL-ECVAM) validated three in vitro methods for developmental toxicity evaluation, namely the rat whole-embryo culture (WEC) using whole rat embryos, the micromass test (MM) employing rodent limb buds and the embryonic stem cell test (EST) based on a permanent mouse embryonic stem cell (mESC) line [13]. Hence, only the EST is free of constant animal use [13,14]. The EST is set up to detect compounds interfering with the development of stem cells into beating cardiomyocytes. This process holds a central role in embryonic development as the heart is the first functional organ in the developing embryo and its performance is central for further developmental progress. Common to these test methods is the rodent nature of test systems.

The predictability of animal-based assays for human health is often limited due to species differences [15,16,17,18,19,20]. During development, general species diversities seem to be smallest during the pharyngula stage [21], yet outside of this short developmental period, rodents and humans differ in developmental tempo [22] and gene regulation programs [23,24]. Additionally, the sensitivity of rodents towards drugs showing adverse effects in humans is poor [20,25]. To overcome species differences, human-induced pluripotent stem cells (hiPSCs) are a well-established alternative and—unlike embryonic stem cells—bear no ethical concerns. Human iPSCs are easily retrieved from donors of any age and sex, representing stem cell characteristics such as self-renewal and pluripotency [26]. Due to this unlimited and ethically inoffensive cell resource, hiPSCs and their biotechnological applications, including spheroids, organoids, and microfluidics, are viewed at the forefront of paradigm-changing 21st-century safety evaluation [27,28,29].

Here we describe a human cell-based alternative test method, the human-induced pluripotent stem cell test (hiPS Test), to study the effects of substances on early embryonic heart development by differentiating hiPSCs into contracting cardiomyocytes mimicking early stages of human development. The test is quick to perform due to the use of hiPSCs in a single-cell culture devoid of fetal bovine serum (FBS). Molecular marker expression as well as positive and negative control establishment using 5-Fluorouracil (5-FU) and Penicillin G (PenG), respectively, indicated the usefulness of the test to assess embryotoxicity.

## 2. Results and Discussion

### 2.1. Characterization of hiPSC Culture for Cardiomyocyte Induction

Human iPSCs are an auspicious tool in a variety of biological research fields as they provide an almost unlimited source of human cells, which are able to differentiate into almost every cell type of the human body. In contrast to embryonic stem cells (ESCs), generating hiPSCs does not require human embryos and therefore does not raise ethical concerns [30]. The research field of developmental toxicology benefits from hiPSCs as they enable in vitro test methods to reduce or even replace animal testing according to the principles of the 3Rs [12,27]. To establish methods that are based on the differentiation of hiPSCs into different cell types, it is necessary to track and document the quality of hiPSC cultures to ensure the reproducibility of high-quality downstream applications [31,32]. The basic culture of hiPSCs, which will be used for differentiation into cardiomyocytes, is of utmost importance for the performance of the hiPS Test. Therefore, we dedicated the first part of the paper to the description of the basic hiPSC culture, highlighting important features of the cells that are critical for performing the hiPS Test.

The commercially available male hiPSC-line iPS11 was used for all experiments conducted. iPS11 cells were banked as a master cell bank (MCB) according to Tigges et al. [32]. A working cell bank (WCB) with single cells cultured in FTDA medium (adapted from Frank et al. [33]) and grown on a Laminin521 (LN521) matrix was generated afterward.

**Figure 1:**
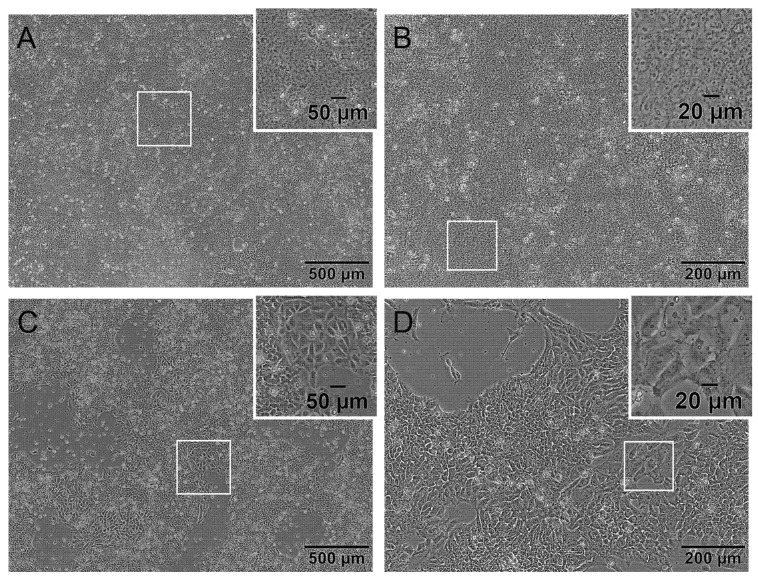
The human induced pluripotent stem cell (hiPSC) line iPS11 grown on Laminin521 (LN521) in FDTA medium displaying pluripotent and differentiated morphology. Representative phase-contrast microscopic images of cultivated iPS11 taken with an Olympus CKX53SF (Tokyo, Japan) and integrated camera SC50. (**A**,**B**): Human iPSCs with a pluripotent morphology indicated by a small cytoplasm to nucleus ratio, multiple nucleoli, and small cell size. (**C**,**D**): Human iPSC culture with differentiated cells showing a higher cytoplasm to nucleus ratio, a larger size, flattened morphology, and differentiation cracks. Magnification 40× (**A**,**C**) and 100× (**B**,**D**).

Cells in culture were quality-controlled by daily microscopic photo documentation. On the day of seeding for the hiPS Test, hiPSCs should have a pluripotent morphology characterized by a low cytoplasm to nucleus ratio, multiple nucleoli, almost no intercellular space and a small cell size shown in Figure 1A,B. Indications of a differentiated state are a high cytoplasm to nucleus ratio, differences in size, a flattened morphology and/or so-called differentiation cracks which are indicated by a white line in the intercellular space seen in Figure 1C,D [33,34].

In this protocol, confluency of 90 to 100% should be reached on day 3 or 4 after cell passaging depending on the initial cell density of 3 × 10^5^ or 2 × 10^5^ cells per 6-well, respectively (Figure S1). Cells should exhibit a morphology according to the images shown in Figure 1A,B. An exceptional day regarding the morphology of the cells is the first day after cell seeding. As cells are passaged as single cells, the commonly used small molecule Y-27632 is added to the medium as a specific inhibitor of rho-associated kinases. It is used to improve the viability of cells during and after passaging. However, using Y-27632 changes the morphology of cells as long as applied seen in Figure S1A,E. Cells build thin fine branches and are not as small as hiPSCs are without Y-27632, which is a normal morphology for day 1 observed in ESC and hiPSC [35,36]. Over the following days, cells regain their typical stem cell morphology (Figure 1A,B and Figure S1D,H).

The evaluation of solely the morphology, however, does not guarantee a successful differentiation into cardiomyocytes. It is generally agreed that hiPSCs should express stem cell markers to a certain extent to be suitable for the application of differentiation protocols. It is suggested that the markers NANOG, OCT3/4, and SOX2, which are intracellular transcription factors, and SSEA4, which is a membrane-bound glycolipid, should at least be expressed to 70% for downstream applications [32,37,38]. Specifically, an OCT3/4 expression of at least 95% has been described to be essential for a successful cardiomyocyte differentiation from hiPSCs [39].

For assessing the expression of these proteins, iPS11 cells were analyzed every other passage from passage 4 to 10, which were used for experiments, as well as one late passage (20) as proof of principle using flow cytometry. The cells were stained with the above-mentioned stem cell markers, ensuring a high-quality base for cardiomyocyte differentiation.

**Figure 2:**
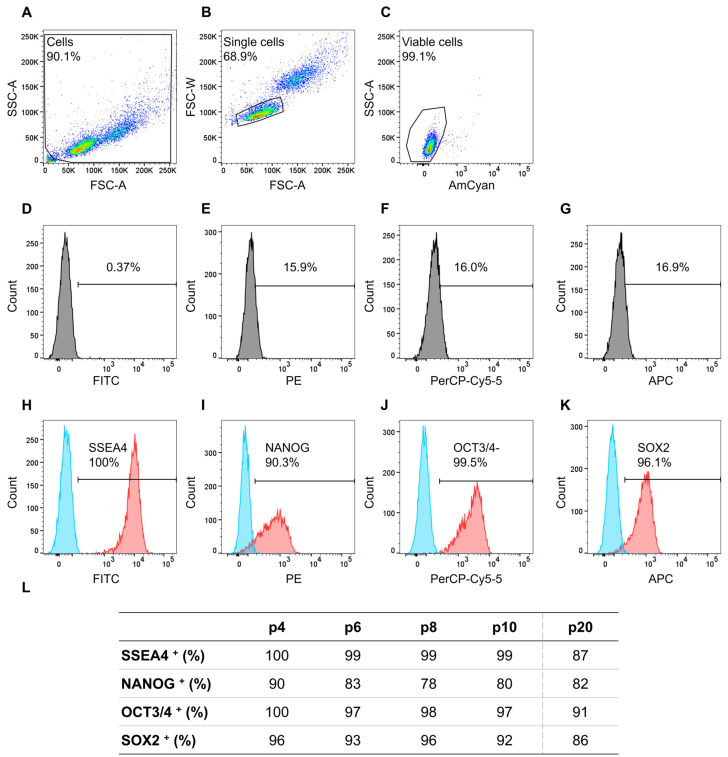
Flow cytometry analyses of cell line iPS11 analyzing stem cell markers NANOG-PE, OCT3/4-PerCP-Cy5.5, SOX2-Alexa Fluor 647, and SSEA-4-FITC, plus fixable viability stain 510 (FVS510) as a live/dead discriminator. The human induced pluripotent stem cell (hiPSC) line iPS11 was cultured in FTDA on Laminin521 (LN521)-coated 6-well plates in a single-cell-based culture and analyzed every other passage (p) from p4 to p10 and p20. The acquisition was performed using a BD FACSCanto™ II system operated with the BD FACSDiva™ software; further analysis was conducted with FlowJo. (**A**): Gating strategy for the relevant cell population. (**B**): Gating strategy to ensure analyses of single cells. (**C**): Gating strategy to discriminate between live and dead cells. (**D**–**G**): Isotype controls for the respective antibodies. (**H**–**K**): The threshold/gate was set to a maximum of 0.49% positive cells in the unstained control, every signal above was counted as a positive signal. Exemplary flow cytometry results of hiPSCs in p4a for the markers NANOG-PE, OCT3/4-PerCP-Cy5.5, SOX2-Alexa Fluor 647, and SSEA-4-FITC, blue peak: unstained cells, red peak: positively stained cells. (**L**): Percentages of positively stained cells (indicated by +) of the flow cytometry analyses from p4 to p10 and p20 for the indicated markers.

Of the gated single cells in Figure 2B, 99% were viable for which further gating was performed. Isotype controls did not show a distinct positive signal (Figure 2D–G) and unstained cells in blue and positive stained cells in red show a distinct distinguishable peak from each other (Figure 2H–K). Results of the flow cytometry analyses for all markers, i.e., SSEA4, NANOG, OCT3/4, and SOX2, and examined passages (p) are listed in Figure 2L. The evaluated stem cell markers consistently reached the threshold of the recommended 70% [32,37,38] from passages 4 to 10. NANOG in p8 had the lowest expression, with 78% of analyzed cells being positive, while all other markers were expressed in 80–100% of the cells. Especially, the iPS11-line met the requirement of 95% positive cells for OCT3/4, identified as crucial for cardiomyocyte differentiation by Lian et al. [39] in all analyzed passages except for p20.

Next, iPS11 cells were differentiated into cardiomyocytes. Video recordings of contracting cardiomyocytes generated from hiPSCs in p4 to p10 and for comparison of p20 are shown in Video S1A–E. While hiPSC differentiations from p4 to p10 of iPS11 consistently resulted in contracting cardiomyocytes covering the entire well (Video S1A–D), a differentiation of p20 hiPSCs did not lead to a confluent contracting structure of cardiomyocytes (Video S1E). Notably, these hiPSCs in p20 did not reach the threshold of the recommended 95% OCT3/4-positive cells (Figure 2L), an instance that might be responsible for the impaired cardiomyocyte differentiation behavior. However, extended cultivation of hiPSCs, in general, is not recommended as cell cultures in higher passages combined with enzymatic passaging are prone to develop chromosomal abnormalities [40,41,42,43]. Therefore, testing for genomic integrity is recommended at least every 12 weeks [44,45] or 15 passages [38,44] during hiPSC culture. In this study, we created an iPS11 WCB from a MCB for cardiomyocyte differentiation according to Tigges et al. [32]. Consecutively, the hiPSCs, which were quality-controlled upon freezing and post-thaw controlled after thawing, were only used for a maximum of 12 passages over a time course of 6 weeks to prevent genomic aberrations. Different methods of hiPSC propagation exist, including colony splitting, which is less prone to genetic instability than single-cell-based expanding. However, the latter is more useful for large-scale experiments [40] and safe to use when combined with profound quality control [32].

### 2.2. Optimization of Cardiomyocyte Differentiation

**Figure 3:**
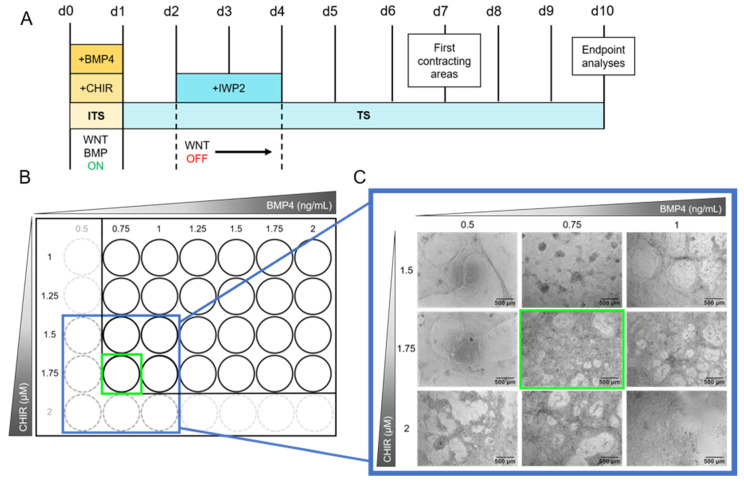
Schematic depiction of the cardiomyocyte differentiation protocol and range-finding experiment of CHIR99021 (CHIR) and bone morphogenetic protein 4 (BMP4) concentrations. (**A**): The protocol starts with the activation of the WNT and BMP signaling pathways by adding CHIR and BMP4, respectively, to the ITS medium. After 24 h, the ITS medium is changed to TS medium. On days 2 and 3 the WNT pathway is inhibited by supplementing TS medium with Inhibitor of WNT Production-2 (IWP2). From day 4 to 10 the medium is replaced every other day with TS medium devoid of pathway modulators. First beating cardiomyocytes are observable on day 7. Endpoint analyses are performed on day 10. For media compositions see Materials and Methods Section 3.2.2 and Supplementary Tables S1–S3. (**B**): The concentrations of CHIR and BMP4 have to be assessed for every hiPSC line individually. Therefore, a grid of 1 to 1.75 µM CHIR and 0.75 to 2 ng/mL BMP4 in increments of 0.25 µM and ng/mL, respectively, was applied using the protocol shown in (**A**), seeding 5 × 10^5^ cells/Matrigel (MG)-coated 24-well in 1.5 mL medium. In case the first results showed an optimal concentration combination at the edge of the grid (green square in (**B**)), the grid was extended framing the optimal concentration (blue square in (**B**)) and tested again. (**C**): Exemplary images of the grid testing for iPS11 cells. The grid was extended to 2 µM CHIR and 0.5 ng/mL BMP4 as shown in (**B**). Concentrations of interest were tested in triplicates seeding 2.75 × 10^5^ cells/MG-coated 48-well following the protocol shown in (**A**) (see Section 3.2.4). Exemplary images were taken on day 10 using an Olympus CKX53SF (Tokyo, Japan) with an integrated camera SC50. Magnification: 40×.

For differentiating the hiPSC line iPS11 into beating cardiomyocytes, the protocol of Zhang et al. [46], which describes a method to differentiate hiPSCs into cardiomyocytes in a 2D culture system by modeling the WNT and BMP signaling pathways, was adapted (Figure 3A). For this purpose, CHIR99021 (CHIR) and bone morphogenetic protein 4 (BMP4) were used to activate the WNT and BMP signaling pathways, respectively.

Cardiomyocyte differentiation from hiPSCs follows two phases of pathway modulation. In the first phase, WNT and BMP pathway activation induces stem cell differentiation into the mesodermal lineage [46]. Thereby, activated WNT initiates *ISL1* expression, a gene necessary for ISL1-positive progenitor cell specification. In the second phase, WNT inhibition due to the addition of IWP2 on day 2 for 48 h (Figure 3A) drives mesodermal cells specifically into the cardiac lineage [47,48]. From day 4 onwards, cells are differentiating further until the first beating areas can be observed on day 7. A continuous, notable beating structure in a wave motion is observed on day 10. (Figure 3A and Video S3). For determining optimal concentrations of CHIR and BMP4 for cardiomyocyte differentiation, these small molecules were titrated in a grid. Such a procedure is always necessary when the basic hiPSC culture protocol undergoes changes, e.g., in the choice of the hiPSC line or the selection of cell culture medium or coating matrix [49]. For a first test, we seeded 5 × 10^5^ cells/well on a 24-well plate (Figure 3B) with increasing concentrations of CHIR (1–1.75 µM) and BMP4 (0.75–2 ng/mL). The optimal concentration combination contained 1.75 µM CHIR and 0.75 ng/mL BMP4 as this culture condition led to the generation of multilayered cardiomyocytes in a mesh structure with holes. As these concentrations are on the edge of the grid, the grid was extended to 2 µM CHIR and 0.5 ng/mL BMP4 and repeated in triplicates seeding 2.75 × 10^5^ cells/48-well (Figure 3C). The optimal concentrations were confirmed resulting in cardiomyocytes beating over the entire well in a notable wave compared to other concentrations (Video S2).

Although the cardiomyocyte differentiation protocol developed by Zhang et al. [45] was the basis for this study, it was adapted accordingly: for higher throughput, we transferred the system from a 24-well to a 48-well format. This adaptation now allows the testing of one substance on a single plate. Furthermore, our protocol uses a TS medium containing 250 µM 2-phospho-L-ascorbic acid, which is of optional use in the protocol of Zhang et al. [46]. To make the protocol more suitable for routine use, we altered the medium change from daily to every other day from day 4 onwards. We titrated the optimal concentrations of 0.75 ng/mL BMP4 and 1.75 µM CHIR in contrast to 1 ng/mL BMP4 and 2 µM CHIR published by Zhang et al. [46] which is probably due to different cell lines used.

### 2.3. Characterization of Cardiomyocytes Generated with the hiPS Test

**Figure 4:**
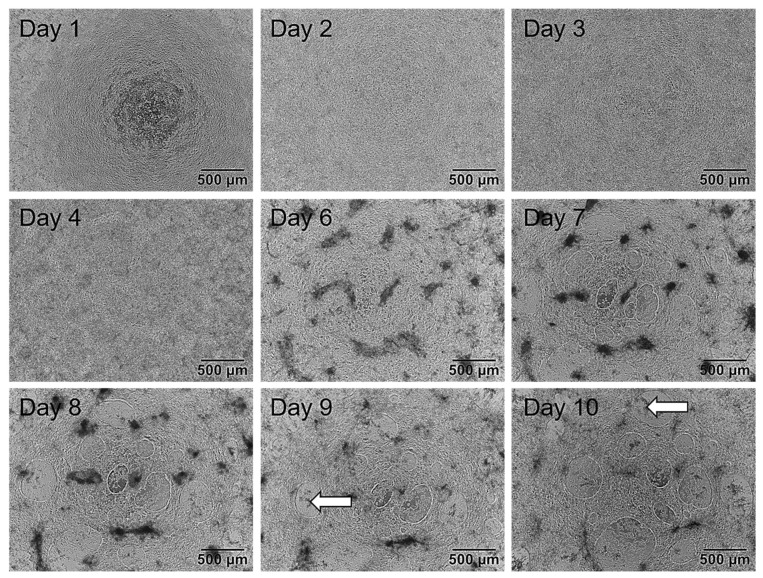
Exemplary phase-contrast images of the same well of human induced pluripotent stem cells (hiPSCs) differentiating into cardiomyocytes over a time course of 10 days. First, 2.75 × 10^5^ cells/Matrigel (MG)-coated 48-well were plated on day 0 in ITS medium. Day 1: Medium was replaced with TS medium. Cells grew on top of each other. Day 2 and 3: Medium was changed to TS supplemented with 2 µM IWP2. Cells grew to uneven heights. Day 4: Medium was changed to TS medium. On day 5 cells were not fed and not tracked. Day 6: TS medium was refreshed. Cells started to form holes and detached from the matrix. Day 7: Cells formed multiple holes and further detachement from the matrix was observed. Areas of beating cardiomyocytes were observable. Day 8: TS medium was refreshed. Cells started to beat in a wave motion. Day 9–10: The morphology of cells did not alter noticeably. The beating developed into a synchronous wave contracting over the entire well on day 10. Dead cells were trapped between the matrix and the beating cells not sucked in during feeding (white arrows). Images were taken with an Olympus CKX53SF (Tokyo, Japan) and an integrated camera SC50. Magnification: 40×.

After evaluating the optimal concentrations of CHIR and BMP4 for the induction of hiPSCs to cardiomyocytes, the hiPS Test was established according to the layout shown in Figure 3A. As a first step, we described the changing morphology of differentiating cells and performed RT-qPCR analyses on each day of differentiation to determine the most suitable time point for endpoint analyses. Morphological features of the cultures displayed typical characteristics on certain days. On day 1 (Figure 4), cells had grown on top of each other indicating loss of cell contact inhibition. After the induction of the mesodermal lineage, cells continued to proliferate and built up multiple layers with uneven levels of height (Figure 4, days 2 and 3). With the inhibition of the WNT pathway by applying IWP2, cell death was a normal phenomenon that can appear stronger on days 3 and 4. Additionally, increased growth of cells on top of each other on day 4 was observed as darker patches (Figure 4, day 4). On day 6 there were denser areas of cells appearing darker on phase-contrast images and the typical mesh with some holes had already started to form, yet cells were still attached to the matrix (Figure 4, day 6). Holes were not always visible on day 6 but appeared on day 7 at the latest (Figure 4, day 7). First contracting areas were observable on day 7 when cells started to lift from the matrix, allowing some areas to beat in a wave motion. At this point, the typical mesh structure consisted of multiple cell layers. From day 8 to 10, the morphology did not change noticeably (Figure 4, day 8–10); however, on day 8 the latest cells would start beating. In the beginning, cells may start beating at multiple points in small waves. On days 9 and 10 of differentiation, not only did the contraction intensity increase but it resulted in a uniform wave motion as shown in Video S3. The beating mesh was connected to the matrix with only a few cell-matrix contacts. Dead cells were trapped under the mesh and did not get sucked up during feeding, staying in place up to day 10 (Figure 4, day 9 and 10, white arrows).

In addition to the morphological characterization, we performed a molecular, RT-qPCR-based analysis of cardiomyocyte differentiation markers on every day of the differentiation protocol. The markers were chosen according to the different stages of cardiomyocyte differentiation and included *OCT4* as a global stem cell marker, the expression of which decreased over time during the differentiation processes [50], *MESP1* as the earliest marker for the cardiac mesoderm [51,52] that is suppressed by WNT-inhibition [53,54], *ISL1* as a marker for multipotent cardiomyocyte progenitor cells that supports survival, proliferation, and migration of the progenitor cells into the primitive heart tube [55,56], *GATA4* as a cardiac progenitor cell transcription factor with a pivotal role in the formation of the linear heart tube that is also expressed in adult cardiomyocytes [57], as well as *TNNT2* and *ACTN2*, which encode for structural sarcomere proteins essential for cardiomyocyte contraction. Of note, in developing cardiomyocytes, *ACTN2* is expressed later than *TNNT2* [58,59].

**Figure 5:**
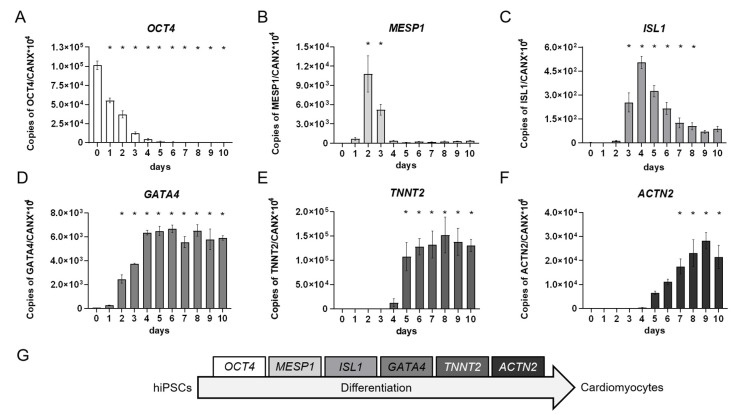
Quantitative real-time RT-PCR analyses of developing human induced pluripotent stem cell (hiPSC)-derived cardiomyocytes over a time course of 10 days. Triplicates of 2.75 × 10^5^ cells/well were seeded onto Matrigel (MG)-coated 48-well plates and differentiated according to the protocol shown in Figure 3A. Each day a triplicate of samples was collected and pooled for RT-qPCR analysis of marker genes for (**A**): stem cells, (**B**): cardiac mesoderm, (**C**,**D**): cardiac progenitor cells, and (**E**,**F**): functional cardiomyocytes. (**G**): Depiction of chosen markers according to the differentiation stages from hiPSCs to cardiomyocytes. Day 0 represents the hiPSCs used for the respective induction collected during the cell passaging procedure. Mean values of copy numbers of the target genes *OCT4*, *MESP1*, *ISL1, GATA4*, *TNNT2*, and *ACTN2* were normalized to the reference gene *CANX* which is stably expressed in hiPSCs and cells of the mesodermal lineage [60,61] including early cardiomyocytes in the hiPS Test (Figure S2). N = 3, ±SEM, *p* < 0.05 was considered significant, * = significant compared to day 0. These gene expression analyses demonstrated the course of different cardiomyocyte developmental stages that differentiating hiPSCs proceed through (Figure 5G) and which were also observed using other differentiation protocols [46,62,63,64,65].

During differentiation, the stem cell marker *OCT4*, as the only gene product expressed on day 0, was strongly downregulated until day 4 of differentiation indicating the loss of pluripotency of the cultures (Figure 5A). Contemporaneously, the expression of the earliest cardiac mesoderm marker *MESP1* arose demonstrating the successful induction of cardiac mesoderm with a peak expression on day 2 (Figure 5B). On day 3, *MESP1* expression decreased and the cardiac progenitor cell marker *ISL1* started to be upregulated. For further cardiomyocyte development, a subsequent decrease in *ISL1* expression is necessary [56], which happened in our cultures from day 4 onwards (Figure 5C). *GATA4*, which is involved in cardiac progenitor cell as well as adult cardiomyocyte function [57], was accordingly upregulated from day 1 onwards and was expressed on a continuous level from day 4 to 10 (Figure 5D). Finally, the cardiomyocyte-specific expression of *TNNT2* and *ACTN2* started after day 4 following inhibition of the WNT-signaling pathway. In agreement with an earlier physiological expression of *TNNT2* compared to *ACTN2* [58,59], we also observed *TNNT2* expression before induction of *ACTN2* (Figure 5E,F). 

**Figure 6:**
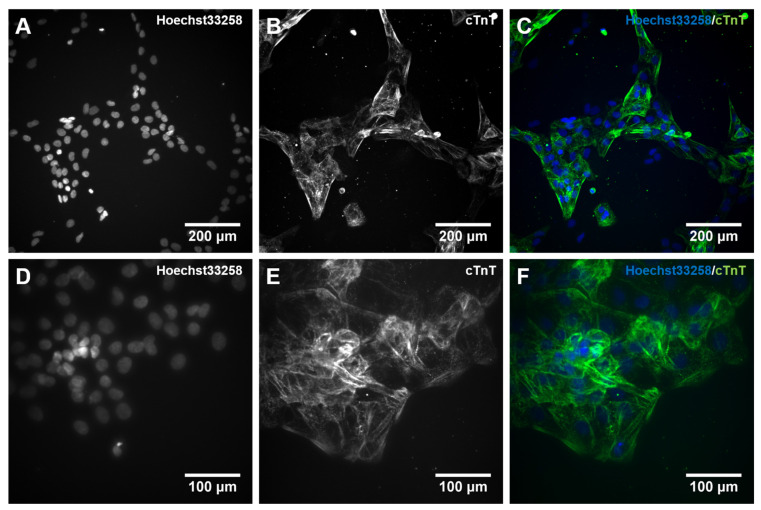
Immunocytochemical (ICC) staining for cardiac muscle Troponin T (cTnT) in 10 days differentiated cardiomyocytes. Human iPSCs were differentiated into cardiomyocytes for 10 days. On day 10, cells were dissociated with Accutase supplemented with 10 µM Y-27632, and 6 × 10^4^ cells were plated on a 96-well plate coated with Matrigel (MG). Cells were cultivated for an additional 24 h and subsequently stained with an antibody against cTnT together with Hoechst 33258. (**A**,**D**): cell nuclei stained with Hoechst33258; (**B**,**E**): cTnT staining, (**C**,**F**): merged pictures. The visualization was performed with an automated microscopic system for high content imaging (CellInsight CX7 LZR Platform). Magnification; (**A**–**C**):100×, (**D**–**F**): 200×.

Following the studies of gene expression during hiPSC differentiation into cardiomyocytes, we qualitatively assessed the protein expression of the *TNNT2* gene product, the cardiac Troponin T (cTnT), using immunocytochemistry (ICC). The staining confirmed the presence of cTnT after 10 days of differentiation. Moreover, it indicated the presence of embryonic cardiomyocytes with immature features such as unorganized sarcomeres missing the typical banding structure (Figure 6B,E). Additionally, stained cells showed no multinucleation (Figure 6C,F), further supporting the immature nature of the cardiomyocytes [66]. Additionally, almost every cell nucleus is attributable to positive staining of cTnT (Figure 6C,F), which supports a high efficiency of the differentiation protocol. Overall, the appearance of these cardiomyocytes is very similar compared to those generated in Zhang et al. [46].

For further characterization and establishment of the hiPS Test method, next we quantitatively analyzed the protein expression of the *TNNT2* and *ACTN2* gene products, the cTnT and α-Actinin2 proteins, respectively, on days 8, 9, and 10 of differentiation using flow cytometry. With these experiments, the proportion of cells expressing cTnT and α-Actinin2 was determined and the optimal time point for endpoint analyses of the hiPS Test was elicited.

**Figure 7:**
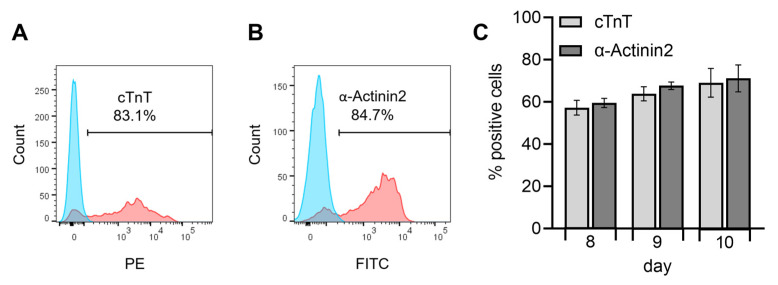
Flow cytometry analysis of human induced pluripotent stem cell (hiPSC)-derived cardiomyocytes analyzing the cardiac-specific proteins cardiac muscle Troponin T (cTnT)-PE and α-Actinin2-FITC, including fixable viability stain 510 (FVS510) as a live/dead discriminator on days 8, 9, and 10 of the differentiation protocol (Figure 3A). Human iPSCs were differentiated into cardiomyocytes in replicates of 16 for every day of analysis and pooled on the respective days. Flow cytometry analyses were performed using a BD FACSCanto™ II system operated with the BD FACSDiva™ software. Further analyses were conducted with FlowJo. (**A**,**B**): Exemplary flow cytometry results of day 10 for the markers cTnT-PE and α-Actinin2-FITC, blue peak: unstained cells, red peak: positively stained cells. The gate to determine a positive signal was defined through the unstained control by setting it to a maximum of 0.49% positively stained cells in the unstained control. (**C**): Results of positively stained cells for the markers cTnT-PE and α-Actinin2-FITC on days 8, 9, and 10. N = 4, ±SEM. *p* < 0.05 was considered significant, no statistical significance was detected.

The gating strategy and controls used for flow cytometry are shown in Figure S3. Unstained cells in blue and positively stained cells in red each show a distinct distinguishable peak exemplarily shown for cardiomyocytes analyzed on day 10 (Figure 7A,B). Specific results for the cardiomyocyte markers displayed a population of cTnT-positive cells of 69 ± 6.8% and α-Actinin2-positive cells of 71.1 ± 6.4% on day 10 of differentiation (Figure 7C). Here the majority of cells were double positive for cTnT and α-Actinin2 and only a small population expressed only one of the markers (Figure S4). Flow cytometry results of day 10 did not differ significantly from the values obtained on day 8 (57.2 ± 3.5% and 59.5 ± 2.2%) or 9 (63.8 ± 3.3% and 67.6 ± 1.8%) for cTnT and α-Actinin2, respectively, yet there is an increasing trend observable over differentiation time (Figure 7C). Zhang et al. [46] reported a generation of 80 to 95% cTnT^+^ cells in 2D for an embryonic stem cell line (HuES6) and three different hiPSC lines (FS3.2, PF6.2, and SPF5.2). The differences in yield might be due to the unknown—and possible later—timepoint of flow cytometry analyses by these authors, or the different stem cell lines used, which are known to frequently affect general hiPSC differentiation outcomes [67,68]. For example, Zhang et al. [46] observed beating generally after 6 days, while iPS11 cells started beating after 7–8 days in culture (Figure 4), a dissimilarity that could be attributed to line or handling differences. The hiPSC differentiation protocols of Lian et al. [39] and Burridge et al. [69], which are also based on WNT and BMP signaling pathway modulation, yielded 90 and 85% cTnT-positive cells, respectively, after 20 days in culture. It is expected that further cultivation of our cardiomyocytes may also reach similar percentages as the number of cTNT-positive cells had not reached a plateau. However, the hiPS Test established here aims at embryotoxicity testing due to disturbance of early cardiac developmental processes. Therefore, the final number of cTNT^+^ cells is not critical for the test as long as a stable expression of cTnT and α-Actinin2 (Figure 7C) and beating of the complete well area, which we attained after 10 days of differentiation (Video S3) is achieved reproducibly between the experiments.

For functional studies, so far microelectrode arrays (MEA) [62,70,71] and visual inspections of beating cardiomyocytes [65,72,73,74] have been performed. Due to only residual attachment of the cells in our 2D protocol, such MEA experiments are inapplicable to this test system as the cells would not reach the electrodes of an MEA chip. To allow a spectator-independent evaluation for the beating behavior, video recordings of the whole well are a valuable alternative that gives information about the entire cell culture. Besides a cell viability assay, comparable test systems often score the presence of beating cardiomyocytes in differentiated embryoid bodies (EBs). However, non-human quantification of the area of contracting cells or effects on the contraction behavior of cardiomyocytes is rarely included in endpoint analyses. MEAs allow for measurement of electrical activity allowing inference to the area of contracting cells. However, this is limited to the number of electrodes on the MEA plates. Furthermore, cells cannot be used for further analysis as fixation or detachment of the cells could damage the electrodes. Therefore, we established a novel method to quantify the beating frequency and area based on video analysis, which allows for further analysis of the cells afterward. The detection and analysis of beating cardiomyocytes directed the assay towards the endpoint analysis on day 10. Beforehand, cells start beating at almost unnotified frequencies synchronizing into a contracting wave until day 10 (Video S3).

Taking the results of the morphological assessment, the RT-qPCR, the ICC staining, the flow cytometry, and the video recordings together, day 10 of differentiation was determined as a suitable time point for the hiPS Test endpoint analyses.

### 2.4. Proof of Concept of the hiPS Test Using an Embryotoxic and Non-Embryotoxic Substance

For further setting up the hiPS Test, we challenged our established protocol with a positive and a negative substance, i.e., 5-FU and PenG. This choice was based on the positive and negative controls used for the previously ECVAM-validated EST [74,75]. 5-FU is a cytostatic drug acting as a pyrimidine analog that is incorporated into RNA or DNA, replacing uracil or thymine, resulting in cell death. In addition, 5-FU is metabolized to fluorodeoxyuridine monophosphate (FdUMP) which can form a complex with the thymidylate synthase inhibiting the deoxythymidine monophosphate (dTMP) production. dTMP is necessary for DNA replication and repair and its depletion leads to cytotoxicity [76].

It was shown that 5-FU induces developmental toxic effects administering it to pregnant rodents [77,78]. Additionally, 5-FU is contraindicated during pregnancy [79]. PenG has been used as an antibiotic against group B streptococcus by pregnant women without showing embryotoxic effects [80,81] and therefore was rendered useful as a negative control compound in the hiPS Test.

**Figure 8:**
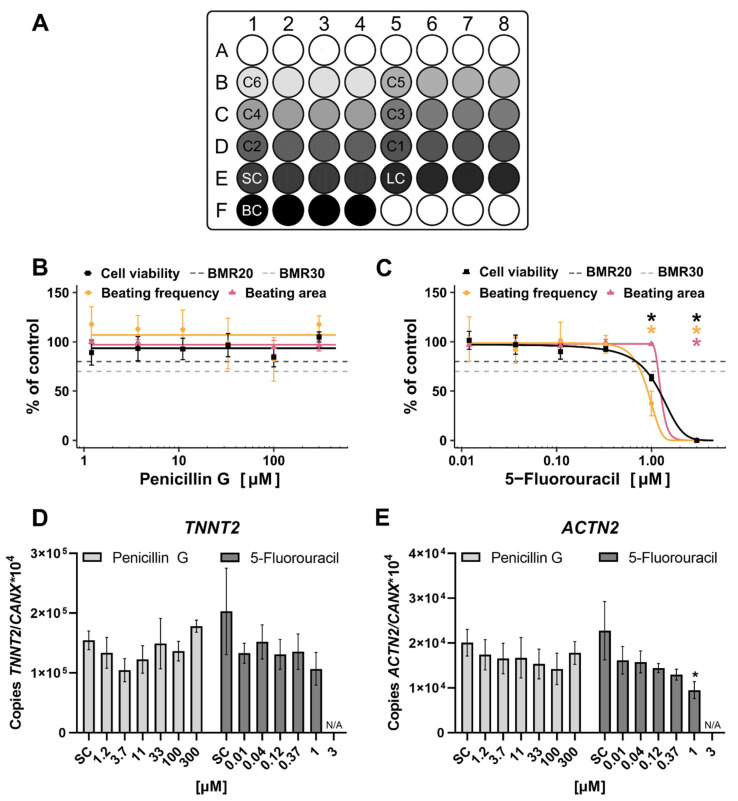
Substance testing of Penicillin G (PenG) and 5-Fluorouracil (5-FU) in the human induced pluripotent stem cell Test (hiPS Test). Human iPSCs were differentiated into cardiomyocytes over a time course of 10 days (Figure 3A). On day 10, endpoints were analyzed. (**A**): Pipetting scheme for compound testing in the hiPS Test. C6-C1: Increasing concentrations of a tested substance in quadruplicates, SC: solvent control, LC: lysis control, BC: background control, white circles: border wells filled with 800 µL sterile water. (**B**–**E**): Cells were treated with 1.2–300 µM PenG or 0.012–3 µM 5-FU for 10 days. Substances were freshly applied on every feeding day (days 2, 3, 4, 6, and 8). (**B**,**C**): Concentration response curves normalized to the control of cell viability, beating frequency, and beating area for PenG and 5-FU, respectively, including the benchmark response (BMR)_20_ and BMR_30_. On day 10 videos of every well were recorded using a Binocular (Leica, Wetzlar, Germany, DS100B) with an integrated heating plate set to 37 °C. Video analyses were performed with the in-house developed software CardioVision to evaluate the beating frequency and beating area. A cell viability assay was performed and analyzed using a multimode-microplate reader (TECAN, Männedorf, Switzerland, Infinite^®^ 200 PRO). (**D**,**E**): Samples were collected (quadruplicates) and pooled for RT-qPCR analysis of *TNNT2*, an early cardiomyocyte marker, and *ACTN2*, a later cardiomyocyte marker. Mean values of copy numbers of the target genes *TNNT2* and *ACTN2* were normalized to the reference gene *CANX* which is stably expressed in hiPSCs and cells of the mesodermal lineage [59,60] including early cardiomyocytes in the hiPS Test (Figure S2). For all experiments, N = 3, ±SEM, *p* < 0.05 was considered significant, * = significant compared to the solvent control. N/A = not applicable.

The plate layout for substance testing in the hiPS Test is shown in Figure 8A. Six concentrations of the respective substance in a serial dilution of 1:3 were tested in quadruplicates.

The scheme follows the suggestions of Crofton et al. [82] designed to identify potential edge effects as the control and the lowest concentrations occupy one well on the edge each which can be compared with the other three replicates not located on the edge. The 5-FU was tested from 3 µM and PenG from 300 µM downwards and the compounds were repeatedly added with every feeding taking place on days 2, 3, 4, 6, and 8. On day 10, endpoint analyses including cell viability, video recordings for analyzing beating frequency and area, and sample collection for qPCR analysis were performed.

The BMC_50_ (benchmark concentration) values of cell viability, beating frequency, and beating area were calculated, which equals the half-maximal inhibitory concentration of viability (IC_50_) and half-maximal inhibitory concentration of differentiation (ID_50_) values, respectively, to better compare our data to other assays’ data.

The results of the two-compound-testing revealed adverse effects of the positive compound 5-FU and no effect of the negative compound PenG in the hiPS Test. PenG did not alter viability, beating frequency, beating area, or cardiac gene expression of differentiating hiPSCs up to 300 µM (Figure 8B,D,E), no BMC_50_ value was reached. Additionally, in the EST PenG did not affect any endpoint up to 2990 µM equaling 1000 µg/mL [74]. Notably, 1000 µg/mL is well above the mean value of the serum concentration of 0.38 ± 0.27 µg/mL (~1 µM) at delivery, measured in pregnant women who took PenG as an antibiotic against group B streptococcus [81]. In the study of Nathan et al. [80] half of the PenG concentration was used which resulted in a serum concentration of 0.14 µg/mL (0.4 µM) after 1 day of injection.

In contrast, 5-FU significantly reduced cell viability, beating frequency, beating area, and *ACTN2* expression. *TNNT2* expression was not altered significantly. Cell viability, beating frequency, and *ACTN2* expression were significantly reduced at 1 µM resulting in a BMC_50_ value of 1.21 µM and 0.92 µM for cell viability and beating frequency, respectively. The beating area was reduced significantly at 3 µM with a BMC_50_ value of 1.25 µM (Table 1; Figure 8C). 5-FU also reduced cell viability in the EST [74] with an IC_50_ of 0.42 µM (Table 1) which does not differ much from the ID_50_ value of 0.36 µM. Performing the same assay but with human ESCs (hESCs) instead of mESCs, called the hESC-based EST, resulted in IC_50_ values of 1.4 and 0.5 µM (Table 1) for the cell lines MRC-5 and H1, respectively [83], which are in the same ballpark as the results of the hiPS Test and EST. An assay substituting mESCs with hiPSCs, called the iPST, resulted in a higher IC_50_ value of 13 µM (Table 1). Here, the ID_50_ value of 4.2 µM is three times lower than the IC_50_ [72]. A 2D assay using hiPSCs called the hiPSC-based EST showed an IC_50_ value of 0.4 nM and an ID_50_ value of 0.018 nM (Table 1) which differ in one order of magnitude.

Comparing the human-cell-based assays among each other the hiPS Test BMC_50_ value of the cell viability was in the same order of magnitude as the IC_50_ value of the hESC-based EST. The hiPS Test is slightly more sensitive compared to the iPST with a difference in BMC_50_/IC_50_ values of one order of magnitude. The hiPSC-based EST, however, is more sensitive than the other assays with an IC_50_ value five orders of magnitude lower compared to the iPST. The same holds true for the ID_50_ value. This might be due to different protocols used for cardiomyocyte differentiation. While Aikawa [72] formed EBs that grow in a 3D structure, Walker et al. [73] chose a 2D protocol resulting in contracting clusters. This leads to a different exposure of cells to the substances tested which might explain the higher IC_50_ and ID_50_ values for 5-FU in the study of Aikawa [72]. Furthermore, different hiPSC lines were used. Additionally, different concentrations of FBS were used in the protocol of Aikawa [72] and Walker et al. [73] (5 and 18%, respectively), which represents a fluctuating variable in cell cultures. The work presented here differentiates cardiomyocytes without EB formation in 2D directly from hiPSCs to cardiac effector cells without using FBS. With this protocol, we aim at reducing variability due to homogenous exposure of all cells to signaling molecules and test substances. The BMC_50_ values for specific endpoints of 5-FU produced with the hiPS Test (0.92–1.25 µM) are in the same ballpark as with the EST (0.36–0.42 µM) [74]. Whether this means that there are indeed limited species differences between mice and humans in cardiac development with regards to the 5-FU mode-of-action cannot be clarified by this work due to the 3D (EST) versus the 2D (hiPS Test) nature of cultures, which might be difficult to compare owing to different in vitro kinetics [84].

**Table 1:** Comparison of half-maximal inhibitory concentration of viability (IC_50_), half-maximal inhibitory concentration of differentiation (ID_50_), and benchmark concentration (BMC_50_) values for 5-FU of in vitro embryotoxicity assays based on stem cell differentiation into cardiomyocytes.

Assay	IC_50_/BMC_50_	ID_50_/BMC_50_
Embryonic stem cell test (EST) [74]	0.42 µM	0.36 µM
hESC-based EST [83]	1.4 µM (cell line MRC-5) 0.5 µM (cell line H1)	X
iPST [72]	13.3 µM	4.2 µM
hiPSC-based EST [73]	0.36 nM	0.018 nM
hiPS Test (this study)	1.21 µM	Beating frequency: 0.92 µM Beating area: 1.25 µM

For compound classification, we used the benchmark dose approach recommended by the EFSA Scientific Committee [85]. For in vitro toxicity testing the term benchmark concentration (BMC) is used instead of benchmark dose, which represents the same calculation principle [86]. The benchmark response (BMR) was determined based on the variability of each evaluated endpoint. For this, the coefficient of variation (CoV) was calculated considering all controls. For cell viability, the CoV was at 16%, therefore, the BMR_20_ was used. For beating frequency and area, the CoV was at 30% and 7%, respectively. While the BMR_30_ was calculated for the beating frequency, the BMR_20_ was calculated for the beating area as 7% is considered extremely low, allowing for a broader range of variability for upcoming compounds to test. The classification model used for the hiPS Test is based on the comparison between the confidence intervals (CI) of the calculated BMC values of the viability and the cardiomyocyte differentiation-specific endpoints. The confidence interval is defined as the range from the benchmark concentration lower limit (BMCL) and the benchmark concentration upper limit (BMCU) given in Table 2. Endpoints are classified as specific (no CI overlap), unspecific (CI overlap ≥ 10%), or borderline (0 > CI < 10%) [87].

No endpoint is classified as a specific hit, as the BMC of every specific endpoint lies within or above the CI of the cell viability (Table 2). Interestingly, the BMCU of the cell viability and the BMCL of the beating area do not overlap, meaning that the viability is reduced although cardiomyocytes still cover the whole well area. As reported above, the beating frequency of hiPSC-derived cardiomyocytes was affected at similar concentrations of 5-FU than mitochondrial activity, the basis for the cell viability assay used (Figure 8B,C). This reduced mitochondrial activity could be the reason for a decreased beating frequency, as cardiomyocytes by nature have a high number of mitochondria to maintain their contracting behavior [88]. Additionally, 5-FU was recently found to decrease mitochondrial membrane potential, increase mitochondrial fragmentation and also decrease the number of cells with a mitochondrial fusion of hiPSCs at 1 µM [89] making adverse effects on mitochondria a possible mode of action for the observed toxicity of 5-FU in vitro. Furthermore, the beating area was not affected at 1 µM, hinting towards a decreased viability, not due to fewer cells but impaired mitochondrial activity. In addition, the significantly decreased expression of *ACTN2* following 1 µM 5-FU exposure with unaffected *TNNT2* expression (Figure 8D,E) points to a stronger effect later in cardiomyocyte differentiation as *ACTN2* is a later cardiomyocyte marker than *TNNT2* [58,59]. Specificity of effects can also be evaluated comparing the IC_50_ of fibroblasts with the IC_50_ and ID_50_ of the cells used for differentiation into cardiomyocytes e.g., mESCs, hESCs, or hiPSCs. This approach is used in the EST, iPST, and hiPSC-based EST, showing that fibroblasts are less sensitive compared to the respective stem cells used.

### 2.5. The hiPS Test Compared to Other Embryotoxicity Assays

One of the earliest crucial features in embryogenesis is heart development. For this reason attention has been focused on this process over the last few decades of alternative method development. To understand their similarities, differences, strengths, and weaknesses, we assembled currently available assays for embryotoxicity evaluation based on cardiomyocyte differentiation in Table 3 and Table 4.

The EST was the first method of its kind producing beating mouse cardiomyocytes through EB formation. It is the only one of such tests that were formally validated by ECVAM. Cells used in the EST are devoid of ethical concerns as these are permanent mouse cell lines (fibroblast cell line 3T3 and mESC line D3). However, there might be species differences in cardiac development between mice and humans [24], which would be unfavorable for the predictive capacity of an in vitro test. The use of FBS in an in vitro method is also undesirable for scientific reasons concerning the non-defined nature and batch variability of serum and the ethical concerns owing to its production [96]. Additionally, the endpoint analysis of scoring contracting cardiomyocytes is relying on the expertise of the staff and the handling time is very high. Therefore, multiple enhancements and additions were developed for the EST to decrease the handling time and add endpoints that were not examined in the original EST to include molecular readouts [75,90,91,92,93,94,95] (Table 3).

To overcome species differences, the hESC-based EST was developed using hESCs instead of mouse cells [83]. Due to the cell material used, this method has been raising ethical apprehensions as working with hESCs underlies restrictions due to ethical issues in many countries worldwide [97,98]. These concerns have been tackled by integrating hiPSCs into the EB-based EST protocol [65,72]. Besides the new cell type, Lauschke et al. [65] introduced an additional endpoint to the former EST protocol, the measurement of EB volumes. Moreover, in contrast to Aikawa [72], Lauschke et al. [65] omitted FBS from their protocol thus proceeding on the track of a true animal-product-free method. Both methods, similar to the original EST, however, need a high number of technical replicates and are thus time and material intensive. Moreover, manual beating analyses require trained staff and are prone to personal bias. According to Zhang et al. [46], who performed a thorough comparison of 2D versus 3D cardiomyocyte differentiation of hESCs and hiPSCs, the 2D method is thought to be superior for generating higher-purity cardiomyocytes. Walker et al. [73] used a 2D method by omitting the step of EB formation and directly differentiating hiPSCs into cardiomyocytes from the monolayer cultures. Instead of a defined medium using WNT and BMP signaling modulation for cardiac differentiation, their protocol employs 18% FBS. In addition, 32 technical replicates are required, even more than those used in the EST (24 replicates) [74]. Such large numbers of replicates are labor intensive to handle, especially when beating behavior is assessed manually. Moreover, they do not allow one compound to be studied on one plate, introducing plate-to-plate variability into one concentration–response curve. One great advantage of the Walker et al. [73] test method over the other assays is the additional molecular readout of cardiac transcription factors *TBX5* and *MEF2c* mRNA expression analyses. Such molecular markers are thought to add sensitivity to the test method. The hiPS Test developed here also uses hiPSCs in a 2D differentiation protocol yet does not include FBS in any medium. The contracting cardiomyocyte structures, albeit in a 2D format, are multilayered and consist of around 70% cardiomyocytes forming a uniform beating area after 10 days in culture. The cell composition of spontaneously differentiating EBs in 3D, as used for the majority of EST protocols, is not defined, generating undefined areas of beating structures. Scoring these partially beating EBs or beating structures in 2D requires experienced staff and cannot be evaluated quantitatively with regards to the beating area so far [13,65,72,73]. The hiPS Test makes use of video recordings fully recognizing all contracting and non-contracting structures. The in-house developed software CardioVision can quantitatively evaluate the percentage of the beating frequency and beating area. This automated evaluation method adds novel opportunities to the evaluation of differentiating cardiomyocytes, making it quantitative in the beating frequency and beating area. This procedure saves time and makes the assay independent of the investigator. Additionally, we established a molecular readout in the hiPS Test. In contrast to Walker et al. [73], who chose *TBX5* expression, an early marker expressed not only in the heart [99], as a readout, we chose the genes *TNNT2* and *ACTN2* that are exclusively expressed in cardiomyocytes and represent more differentiated stages of cardiomyocyte development. Furthermore, the hiPS Test gets by with only four technical replicates and is performed on a 48-well plate. Due to the single-cell passaging, hiPSCs can be propagated very quickly. This enables a higher throughput of the hiPS Test compared to assays using EBs. However, the method also has a serious drawback. The cardiomyocyte differentiation itself is very sensitive to fine disturbances. Here, the basic culture is crucial. Not only the quality of the hiPSCs but also the cell density before plating both play major roles. Only subtle deviations lead to unsuccessful differentiation. Similarly, the FTDA medium composition is crucial. Due to its composition of a large variety of different single components, it is highly error prone. Practically in the lab, this sensitivity leads to either successful differentiation or no differentiation over the whole plate, hence not affecting intra-plate variability. Due to this circumstance, we currently optimize the medium for a more stable and reproducible method that will enhance the hiPS Test’s practical applicability and will enable us to screen a panel of test substances.

## 3. Materials and Methods

### 3.1. Compounds

The 5-FU (#F6627) and Penicillin G sodium salt (#PENNA) were obtained from Merck (Darmstadt, Germany). The 5-FU stock solution (10 mM) was prepared in DMSO (Carl Roth, Karlsruhe, Germany, #A994.1), PenG (1 M) was dissolved in sterile MilliQ water.

### 3.2. Cell Culture

The hiPSC-line iPS11 was purchased from Alstem (Richmond, CA, USA, #iPS11). Cells were retrieved from human foreskin fibroblasts and transfected footprint free by ectopic expression of OCT4, KLF4, and L-MYC using Alstem episomal plasmids. To ensure a high-standard cell line to work with, we used a two-tiered banking process, producing a fully characterized MCB and a partially characterized respective WCB generated according to Tigges et al. [32]. Characterization included the study of morphology, mycoplasma contamination, cell line identity, karyotype stability, cell antigen expression and viability, gene expression, pluripotency, and post-thaw recovery.

#### 3.2.1. Coating of Cell Culture Dishes

For the hiPSC culture, 6-well plates (Sarstedt, Nümbrecht, Germany) were coated with LN521 (BioLamina, Sundbyberg, Sweden, #LN521-05) as described in Tigges et al. [32].

For cardiomyocyte differentiation, 48-well plates (Greiner Bio-One, Kremsmünster, Österreich) were coated with MG (Corning, Corning, NY, USA, #354263). Beforehand, a 10 mL serological pipet, 48-well plates, and a 5 mL Combitip^®^ were precooled at −20 °C. MG was initially diluted 1:3 in KnockOut™ DMEM (KODMEM; Thermo Fisher SCientific, Waltham, MA, USA, #10829018) and frozen at 1 mL aliquots at −20 °C. For coating of 48-well plates, one 50 mL conical tube with 24 mL of cold KODMEM and two with 36 mL KODMEM were placed on ice. One MG aliquot was dissolved in 6 mL out of the 24 mL cold KODMEM by continuously pipetting up and down using the precooled 10 mL serological pipet. After dissolving the aliquot, the solution was transferred back resulting in a 1:75 dilution. Next, 12 mL of the MG solution were each transferred to the conical tubes containing 36 mL KODMEM and mixed thoroughly resulting in a 1:300 dilution. For coating, 150 µL/48-well of the 1:300 MG solution was pipetted using a Multipette^®^ M4 and the precooled 5 mL Combitip^®^. Plates were sealed with parafilm and incubated overnight at room temperature (RT). After an additional 24 h at 4 °C plates could be used for up to 4 weeks stored at 4 °C. Before seeding cells, plates were equilibrated to RT for at least 15 min. Cells were directly seeded onto the matrix after aspirating the coating solution.

For immunocytochemistry, wells of a 96-well plate (Greiner Bio-One, Kremsmünster, Österreich) were coated with 50 µL of the 1:300 MG solution. For range-finding experiments in a 24-well plate (Sarstedt, Nümbrecht, Germany), 250µL/well of the 1:300 MG solution was used.

#### 3.2.2. Cell Culture Media

All media were prepared under aseptic conditions. FTDA and TS medium were stored at 4 °C for up to 4 weeks, ITS medium was prepared freshly for every induction. For feeding, appropriate amounts were warmed to 37 °C to avoid repeated heating phases.

##### FTDA Medium

Human iPSCs were cultivated in FTDA (FGF, TGF, Dorsomorphin, ActivinA) medium, which is composed of DMEM/F12-L-glutamine, with the following supplements; 1% Lipid Mix, 0.1% Human serum albumin, 2 mM L-Glutamine, 100 U/mL Penicillin/0.1 mg/mL Streptomycin, 50 ng/mL animal-free recombinant human (rh) FGF-basic 154 a.a., 1:1000 ITS Premix Universal Culture Supplement resulting in 5 ng/mL Insulin, 5 ng/mL Transferrin and 5 pg/mL Selenious acid, 4 ng/mL rhActivinA, 0.2 ng/mL TGFβ1, and 50 nM Dorsomorphin. For detailed information see Table S1.

##### ITS Medium

ITS medium was used for the initial induction of hiPSCs into cardiomyocytes on day 0. It is composed of KODMEM, with the following supplements: 2 mM L-Glutamine, 100 U/mL Penicillin/0.1 mg/mL Streptomycin, 1:1000 ITS Premix Universal Culture Supplement resulting in 5 ng/mL Insulin, 5 ng/mL Transferrin and 5 pg/mL Selenious acid, 10 µM Y-27632, 25 ng/mL animal-free rhFGF-basic 154 a.a., 0.75 ng/mL rhBMP4, and 1.75 µM CHIR99021. For detailed information see Table S2.

##### TS Medium

TS medium was used for the maintenance of cardiomyocytes from day 1 onwards. It was composed of KODMEM with the following supplements: 2 mM L-Glutamine, 100 U/mL Penicillin/0.1 mg/mL Streptomycin, 1:100 TS mix resulting in 5.5 µg/mL hTransferrin and 6.7 ng/mL sodium selenite, and 250 µM 2-Phospho-L-Ascorbic Acid. For detailed information see Table S3.

#### 3.2.3. hiPSC Culture

##### Thawing of hiPSCs

One cryovial of the WCB contained 1.5 million cells. For thawing, the cryovial was taken out of the nitrogen tank and warmed in a water bath until a pea-sized clump of frozen cells was left. The thawed solution was transferred to a 15 mL conical tube. A volume of 1 mL of FTDA (4 °C) was added dropwise to the thawed cell solution and the conical tube was shaken after each drop to ensure equilibration of the cells to the new external milieu. Afterward, 1 mL of cold FTDA was added to the cryovial to take up the remaining cells. This solution was also transferred dropwise to the conical tube, which was again shaken after each drop. Additional 2 mL of cold FTDA were added to the conical tube slowly. Cells were centrifuged at 200 g for 5 min at RT. The supernatant was removed with a vacuum pump and cells were resuspended in 4 mL FTDA (37 °C) supplemented with 10 µM Rock-Inhibitor Y-27632 (HelloBio, Bristol, UK, #HB2297). 2 mL per LN521-coated 6-well were seeded. After 24 h the cell culture was treated as described in “Cell passaging”. Cells were passaged 3 times before experiments were conducted to ensure a full recovery after thawing.

##### Cell Passaging

Cells were split twice a week when 90–100% confluency was reached. For passaging, hiPSCs were washed once with PBS without Mg^2+^ and Ca^2+^ (-/-; Merck, Darmstadt, Germany, #D8537) and dissociated enzymatically using 1 mL Accutase (Thermo Fisher Scientific, Waltham, MA, USA, #A11105-01) supplemented with 10 µM Y-27632 per 6-well for 12 min at 37 °C and 5% CO_2_. Afterward, Accutase was diluted by adding 2 mL/6-well of DMEM-F12-L-Glutamine (Thermo Fisher Scientific, Waltham, MA, USA, #21331020), and cells were collected in a 50 mL conical tube. Cells were resuspended 10 times using a 10 mL serological pipet to ensure a single-cell suspension which was then counted in a Neubauer improved chamber. 2 or 3 × 10^5^ cells/6-well depending on the days until 90–100% confluency was supposed to be reached (4 and 3, respectively), were centrifuged at 200× *g* for 2 min at RT. The supernatant was aspirated using a vacuum pump and cells were resuspended in 2 mL/6-well FTDA supplemented with 10 µM Y-27632 and plated on an LN521-coated 6-well plate (see methods part Section 3.2.1). Note that exposure to 10 µM Y-27632 should not exceed 24 h. Cells were further cultivated in FTDA without Y-27632. Human iPSCs were supplied with an additional 0.5 mL FDTA for every day in culture (2.5 mL/6-well on the 1st day after cell passaging, 3 mL/6-well on the 2nd day after cell passaging, etc.). When hiPSCs were in culture for 4 days, cells were passaged as described above. After at least 2 h cells were fed with an additional 2 mL without Y-27632 to guarantee the survival of the prolonged period (1 day) without medium replacement. An induction into cardiomyocytes can only be performed when cells were in culture for 3 days; otherwise, the efficiency of the induction is reduced. Confluency of 90–100% is critical for the induction as well (Figure S1).

#### 3.2.4. Cardiomyocyte Differentiation

Cardiomyocyte differentiation was performed as described in Zhang et al. [46] for 2D cell cultures with minor changes described hereafter (see also Figure 3A). As mentioned above, induction was performed when hiPSCs were cultured for 3 days and reached a confluency of 90–100%. First, the medium of desired wells was refreshed with 2 mL FTDA/6-well. After at least 2 h cells were treated for cell passaging as described in “Cell passaging”. After counting, 2.75 × 10^5^ cells/MG-coated 48-well were centrifuged at 200× *g* for 2 min at RT. The supernatant was aspirated with a vacuum pump and cells were resuspended and plated in 800 µL/48-well in ITS medium (day 0; Table S2). After 24 h ITS medium was completely aspirated with a vacuum pump and replaced with TS medium (day 1; Table S3). On days 2 and 3 medium was changed to TS medium supplemented with 2 µM IWP2 (Tocris, Bristol, UK, #3533). On day 4, medium was replaced with TS medium and changed every other day until day 10. Of note, from day 4 onwards medium has to be changed carefully by manual pipetting as the cells start detaching from the matrix and get sucked in easily when using an aspiration system. For substance testing, the compound was supplemented to the respective medium and thus applied freshly every time the medium was changed.

### 3.3. Cardiomyocyte Beating Analysis

On day 10, before recording the videos, cells were fed with 600 µL of TS medium to ensure an equal amount of nutrition supply, followed by a 2 h incubation at 37 °C and 5% CO_2_ to allow floating particles to settle. Afterward, the plate was placed on a heating plate integrated into the binocular Leica DMS1000 B (Wetzlar, Germany) set to 37 °C. The lid of the respective 48-well plate was removed to ensure a high quality of recorded videos. The plate was equilibrated for 10 min to the ambient environment. Videos were recorded for 10 sec or at least until the occurrence of two contractive motions using the binocular (magnification 1×). Beating frequency and beating area were analyzed using the in-house-developed software CardioVision (Dönmez et al. in preparation).

**Figure 9:**
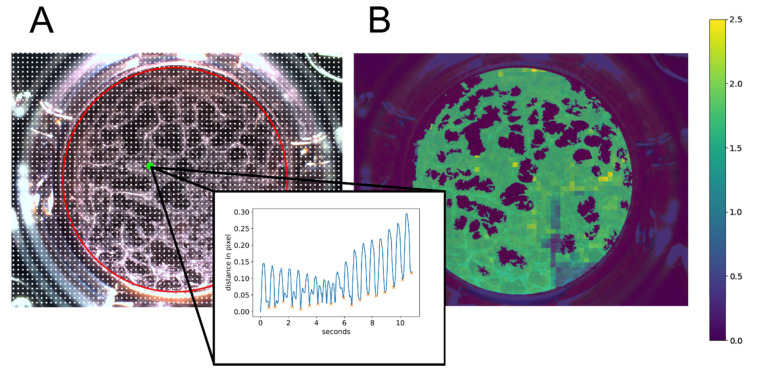
Visualization of video processing steps used in the in-house developed software CardioVision. The software includes different video processing steps to finally determine the beating frequency and beating area of a recorded video. (**A**): Reference points (white dots) were placed over the video in a grid of 20 pixels to each other. Reference points within the region of interest (ROI), depicted as a red circle, were analyzed. The motion profile of a single reference point marked in green is depicted inside the black frame. Peaks marked with an orange X are counted as a beat. (**B**): Heatmap representing the beating area color-coded with the respective beating frequency in beats/second calculated at each reference point.

Briefly, the following steps were applied to each video. First, the motion profile was determined. For this, reference points were placed on the video as a grid with a distance of 20 pixels to each other (Figure 9A, white dots). For every reference point, the motion was tracked using the Lukas Kanade function, provided in the OpenCV library [100], evaluating the function frame by frame. As every motion is tracked in this step, it is crucial to further distinguish between the motion of cells and artifacts, e.g., floating particles. Each reference point’s motion creates a distance profile which reflects how far the reference point moved from the starting point (first frame) at time t (Figure 9A). A threshold alpha was applied on the calculated distance from one frame to another for each reference point to exclude extremely small distances. This threshold enables a noise reduction for the analysis.

Next, a region of interest (ROI) reflecting the total area of the well is determined with a padding of 10 pixels (Figure 9A, red circle) performing the Hough transformation using the ‘HoughCircles’ function of the OpenCV library [100].

To distinguish between the cell layer and dead cells or small artifacts, a cell mask was created. The first frame of a video was converted to HSL (hue, saturation, lightness) color representation. Afterward, the mean value of lightness was calculated within the ROI. Based on the average lightness, thresholds were assigned to certain value ranges. This is used to optimize the binarization of the ROI to predict the cell layer. After binarization, connected components were determined using the OpenCV function ‘connectedComponentsWithStats’ excluding small areas which represent dead cells or artifacts. Thus, the cell mask is the upper bound for further analysis of the beating area and beating frequency. The endpoint beating frequency was determined by performing peak analyses on the distance profile curves of the reference points within the cell mask. The result was visualized by creating a heat map (Figure 9B, using color codes for the individual beating frequency results. The total area of the beating frequency represents the beating area.

### 3.4. Cell Viability Analysis

Cell viability was assessed using the CellTiter-Blue^®^ Cell Viability Assay (CTB; Promega Corporation, Madison, WI, USA, #G8081). The CTB reagent was mixed 1:3 with TS medium and warmed to 37 °C. After recording the videos as described in 3.3, lysis control wells were treated with 0.2% Triton X-100 (Merck, Darmstadt, Germany, #T8787; diluted in dH_2_O) for 20 min at 37 °C and 5% CO_2_. Afterward, 200 µL of the CTB mixture was added to each well. Plates were incubated for 1 h at 37 °C and 5% CO_2_, before analysis. The plate was analyzed using a multimode-microtiter plate reader (Tecan, Männedorf, Switzerland, Infinite M200Pro) at an A540/590 ratio.

### 3.5. Quantitative Reverse-Transcription qPCR

For the time point ‘day 0′, 1–2 million hiPSCs were collected during the cell passaging procedure of the respective experiments. For characterization (Figure 5), cell lysates of 3 technical replicates were collected and pooled for every day from day 1 to 10 of differentiation. For PCR analysis of treated cells (Figure 8), all 4 replicates were collected and pooled on day 10. For all experiments, RNA was isolated using the peqGOLD Total RNA Kit (VWR, Radnor, PA, USA, #13-6834-02P) including a DNA digestion step with the DNase I Digest Kit (VWR, Radnor, PA, USA, #12-1091-02) according to the manufacturer’s protocol. For cDNA synthesis, RNA concentration was measured using a NanoDrop™ (Thermo Fisher Scientific, Waltham, MA, USA) microvolume spectrophotometer. For one sample, 110 ng of RNA was mixed with 1.25 µL Oligo (dT)_15_ Primer (100 µM) and 1 µL dNTP Mix (10 mM; Jena Bioscience, Jena, Germany, #PM303L and #NU1006S, respectively) and annealed at 64 °C for 5 min in a T3 Thermocycler (Biometra, Jena, Germany). Afterward, the reaction mixture, together with 5 µL RNase free water, 4 µL RT reaction buffer (5×), and 1 µL M-MLV Reverse Transcriptase (200 U/µL, Promega Corporation, Madison, WI, USA, #M1705) were used according to the manufacturer’s instructions and heated to 37 °C for 52 min and 70 °C for 15 min in a T3 thermocycler. Quantitative RT-PCR was performed using the QuantiFast SYBR^®^ Green PCR Kit (Qiagen, Hilden, Germany, #204056) operated using a Rotor Gene Q cycler (Qiagen, Hilden, Germany) according to the manufacturer’s instructions. For quantitative analysis, standards were used to calculate copy numbers of the genes of interest normalized to copy numbers of the reference gene *CANX*, which was proven to be stably expressed in hiPSCs differentiating into cells of the mesodermal lineage [60,61] including early cardiomyocytes in the hiPS Test (Figure S2). The procedure was performed as described in Dach et al. [101] with the following changes. The purified DNA was measured using a NanoDrop™ microvolume spectrophotometer and the numbers of molecules/µL were calculated. A stock solution of 3.75 × 10^10^ molecules/µL per gene of interest was generated and stored at 4 °C for a maximum of two weeks. 1:10 dilutions from 3.75 × 10^2^ up to 3.75 × 10^7^ molecules/µL were quantified with three independent runs of RT-qPCR as stated above. Primer sequences are listed in Table S4.

### 3.6. Flow Cytometry Analysis

All flow cytometry analyses were performed using a BD (Franklin Lakes, NJ, USA) FACSCanto™ II system operated with the BD (Franklin Lakes, NJ, USA) FACS Diva Software Version 6.1.3. For all experiments at least 10,000 events per condition were recorded from the scatter gate. Further analysis was performed using FlowJo V10.7.1 (Franklin Lakes, NJ, USA). For the preparation of cells, all steps were performed at RT unless stated otherwise.

#### 3.6.1. hiPSCs Culture

To evaluate stem cell markers on protein level, we performed flow cytometry using the Human Pluripotent Stem Cell Transcription Factor Analysis FACS Kit (BD, Franklin Lakes, NJ, USA, #560589) and extended the kit with the glycolipid SSEA4 as described in Tigges et al. [32]. All markers used are listed in Table 5. To discriminate between dead and live cells the Fixable Viability Stain (FVS) 510 (BD, Franklin Lakes, NJ, USA, #564406) was added to the setup.

Cells were analyzed every second passage from p4 to p10 and p20 at 90–100% confluency. Human iPSCs of five 6-wells were dissociated as described in ”Cell passaging” and counted in a Neubauer improved chamber. The staining was performed as described in Tigges et al. [32]. Briefly, 1.2 × 10^6^ cells per staining condition were transferred into a 1.5 mL Eppendorf reaction tube. Cells were washed with 500 µL PBS -/- once and resuspended in 500 µL FVS 510 diluted 1:1000 in PBS -/- for 15 min. The unstained sample was resuspended in 500 µL stain buffer (BD, Franklin Lakes, NJ, USA, #554656) instead. Then, cells were washed twice with 1 mL stain buffer (centrifugation at 300× *g* for 5 min) and samples were stained with 100 µL SSEA-4 antibody or isotype control diluted in Perm/Wash buffer (1:6) for 25 min. Cells were washed twice with 1 mL PBS -/- and fixed with 300 µL BD Cytofix Fixation Buffer (BD, Franklin Lakes, NJ, USA, #554655) for 20 min. Cells were washed twice with 1 mL PBS -/- and stored in PBS -/- at 4 °C overnight. Centrifugation steps were performed at 500 g for 5 min after the fixation step. After centrifugation, cells were resuspended in 300 µL BD Perm/Wash Buffer (BD, Franklin Lakes, NJ, USA, #554723) for 15 min. Cells were centrifuged and resuspended in the respective antibodies and isotype controls diluted in Perm/Wash Buffer (1:6) for 30 min. Cells were washed twice with 1 mL Perm/Wash Buffer and then resuspended in 300 µL stain buffer for analysis.

#### 3.6.2. Human iPSC-Derived Cardiomyocytes

Differentiated hiPSC-derived cardiomyocytes were analyzed for the expression of the cardiomyocyte-specific markers cTnT and α-Actinin2 on days 8, 9, and 10. For every day of analysis 16 wells of a 48-well plate were pooled. Briefly, the medium was removed and 300 µL Accutase/48-well was added for 7 min at 37 °C and 5% CO_2_. Cells were resuspended afterward using a 1000 µL pipet to ensure a singularization and placed at 37 °C and 5% CO_2_ for an additional 3 min. The cells of 16 wells were pooled into a 50 mL conical tube and wells were washed once with 300 µL PBS -/- which was also added to the conical tube. 2.4 mL DMEM-F12-L-Glutamine were added to the cell suspension to further dilute the Accutase. Cells were resuspended with a 5 mL serological pipet 15 times to ensure a single-cell suspension. The suspension was distributed evenly into 6 Eppendorf reaction tubes for the following staining conditions: (1) unstained, (2) FVS 510 (1:1000) only, (3) Isotype controls; Mouse IgG1, κ (BD, Franklin Lakes, NJ, USA, #550617; 1:334) and REA Control Antibody (I), human IgG1, FITC, REAfinity™ (Miltenyi Biotec, Bergisch Gladbach, Germany, #130-118-354; 1:50) + FVS 510 (1:1000), (4) single staining with PE-Mouse Anti-Cardiac Troponin T (BD, Franklin Lakes, NJ, USA, #564767; 1:20) + FVS 510 (1:1000). (5) single staining with α-Actinin2 (Sarcomeric) Antibody, anti-human/mouse/rat, FITC, REAfinity™ (Miltenyi Biotec, Bergisch Gladbach, Germany, #130-119-806; 1:50) + FVS 510 (1:1000), (6) all antibodies + FVS 510 (1:1000). Cells were centrifuged at 300× *g* for 5 min at 4 °C and washed once with 500 µL PBS -/-. Afterward, samples 2–6 were resuspended in 500 µL PBS -/- + FVS 510 (1:1000) and incubated for 30 min at 4 °C. Sample 1 was resuspended in 500 µL PBS -/- alone. Cells were washed twice with 500 µL stain buffer and fixed for 20 min in 500 µL of Cytofix Fixation Buffer. Cells were washed twice (500 g, 5 min) with 500 µL PBS -/- and stored in PBS -/- at 4 °C overnight. The next day, cells were centrifuged at 500× *g* for 5 min and resuspended in 500 µL Perm/Wash buffer. After 5 min of incubation, this step was repeated. Afterward, cells were resuspended in 100 µL of the respective antibodies and isotype controls each diluted in Perm/Wash buffer and incubated for 30 min. Then, cells were washed twice with 500 µL of Perm/Wash Buffer. Afterward, cells were resuspended in 300 µL stain buffer for analysis.

### 3.7. Immunocytochemistry

On day 10, cells were dissociated as described in 3.2.3.2. For replating, 6 × 10^4^ cells were seeded onto MG-coated 96-well plates in 200 µL TS medium/well for 24 h. For fixation, 50 µL medium was removed and 50 µL 12% paraformaldehyde (PFA; Merck, Darmstadt, Germany, #P6148) was added resulting in a 4% PFA-solution. Cells were incubated for 30 min at 37 °C. Afterward, cells were washed three times with 300 µL PBS -/-. For permeabilization, 50 µL/well of 0.1% Triton-X 100 in PBS -/- was applied for 5 min and cells were washed once with 300 µL PBS -/-. A blocking step was performed with 50 µL/well of 10% goat serum (Merck, Darmstadt, Germany, #G9023) in 0.1% Triton-X 100 for 30 min at 37 °C. Afterward, the antibody solution (50 µL/well) consisting of 1:200 Mouse Anti-Cardiac Troponin T antibody [1C11] (Abcam, Cambridge, UK, #ab8295) and 10% goat serum in 0.1% Triton-X 100 was applied for 60 min at 37 °C. Three washing steps were performed as described above. The second antibody solution (50 µL/well) consisting of 1:200 Goat anti-Mouse IgG (H + L) Cross-Adsorbed Secondary Antibody, Alexa Fluor 488 (Thermo Fisher Scientific, Waltham, MA, USA, #A-11001), 2% goat serum, and 1% Hoechst33258 (Merck, Darmstadt, Germany, #B1155) in PBS -/- was added to the wells for 30 min at 37 °C. Finally, the wells were washed three times with 300 µL PBS -/-. Fluorescence imaging was performed using an automated microscope system used for high content imaging (CellInsight CX7 LZR Platform, Thermo Fisher Scientific, Waltham, MA, USA).

### 3.8. Data Analyses and Statistics

Statistical analyses were performed with GraphPad Prism 8 for Windows 64-bit, version 8.4.3 (San Diego, CA, USA). All data are presented as the mean of ≥3 N ± SEM analyzed with a one-way ANOVA followed by a Dunnett multiple comparisons test; *p* ≤ 0.05 was considered to be significant.

The best curve-fitting model was chosen automatically from a large pool of 13 mathematical concentration–response functions according to robust statistical criteria. Fitted curves can follow linear, sigmoidal, monotonic, and non-monotonic trends. The estimation method assumes continuous response data. Statistical computing is based on the Loglik R package and ecosystem, a generic, object-oriented, and extensible framework for analysis of in vitro data for the R language that relies on the established R package drc [102].

### 3.9. Software

For phase-contrast microscopy, CellSens Entry (Hamburg, Germany) version 1.18 was used. BD (Franklin Lakes, NJ, USA) FACSDiva™ (6.1.3) was used to operate the BD (Franklin Lakes, NJ, USA) FACSCanto™ II system. FlowJo (Franklin Lakes, NJ, USA) version 10.7.1 was used for further analysis of flow cytometry data. The TECAN multimode-microplate reader was operated with the software i-control™ (Männedorf, Schweiz) version 3.22. Rotor-Gene Q series (Hilden, Germany) version 2.3.4) was used to operate the Rotor Gene Q PCR-cycler and to analyze the results of the RT-qPCR. Cardiomyocyte beating analysis was performed with the in-house developed software CardioVision described in Section 3.3.

## 4. Conclusions

We established the hiPS Test for contributing to a future test battery of in vitro embryotoxicity tests [10]. While we made very good progress in setting up the test method up in a 48-well plate format, allowing us to study one compound per plate, in automated video analyses for assessing beating frequency and area, as well as molecular analyses using qPCR, cell culture medium optimization for enhancing the stability of the protocol will augment its practical use.

## Figures and Tables

**Table 2:** Benchmark concentration (BMC) values including the Benchmark concentration lower limit (BMCL) and Benchmark concentration upper limit (BMCU) calculated with the respective Benchmark response (BMR) for the substance 5-FU.

Endpoint	BMC in µM	BMCL–BMCU in µM
Cell viability	BMC_20_ 0.69	0.37–0.94
Beating frequency	BMC_30_ 0.77	0.33–0.99
Beating area	BMC_20_ 1.14	1.02–2.11

**Table 3:** Past additions and enhancements to the classical embryonic stem cell test (EST) [74] to improve the test system.

	Addition/Enhancement	Advantage Compared to Classical EST
Pellizzer et al. [90]	RT-PCR	Additional endpoint
Peters et al. [91]	Embryoid body (EB) formation in 96-well plate Introduction of Relative Embryotoxic Potency (REP) values	Higher throughput
Peters et al. [92]	Automated contractility assessment	Minimizing experimenter-dependent evaluation
van Dartel et al. [93]	Transcriptomics	Additional endpoint
Seiler and Spielmann [75]	Molecular marker analysis using flow cytometry	Additional endpoint
Suzuki et al. [94]	Evaluation of luciferase activity of reporter cell lines for *HAND1* and *CMYA1*	Additional endpoint
Witt et al. [95]	Automation of cell seeding, compound dilution and transfer, media exchange, and cell viability assay, EB transfer to imaging plates Marker expression analysis by imaging	Higher throughput Additional endpoint

**Table 4:** Published in vitro embryotoxicity assays based on stem cell differentiation into cardiomyocytes.

Assay	Species	Cell Type	2D/3D	Endpoints	Advantages	Disadvantages
Embryonic stem cell test (EST) [74]	Mouse	Mouseembryonic stem cells Mouse fibroblasts	3D	Cell viability Beating evaluation (yes/no principle)	3D cell system ECVAM validated	Rodent cells FBS in medium Low throughput due to high number of technical replicates Experienced staff needed for visual evaluation of beating
hESC-based EST [83]	Human	hESCs Human embryonic lung fibroblasts	3D	Cell viability	3D cell system	FBS in fibroblast medium Low throughput due to high number of technical replicates Ethical concerns using hESCs Only viability evaluation
iPST [72]	Human	hiPSCs Adult human dermal fibroblasts	3D	Cell viability Beating evaluation (yes/no principle) as a function of time	3D cell system No ethical concerns using hiPSCs	FBS in medium Low throughput due to high number of technical replicates Experienced staff needed for visual evaluation of beating
Pluri Beat Assay [65]	Human	hiPSCs	3D	Cell viability Beating evaluation with ‘beat score’ EB volume	3D cell system No FBS No ethical concerns using hiPSCs	Low throughput due to high number of technical replicates Experienced staff needed for visual evaluation of beating
hiPSC-based EST [73]	Human	hiPSCs Human foreskin fibroblasts	2D	Cell viability Beating evaluation (yes/no principle) mRNA expression	No ethical concerns using hiPSCs	FBS in medium Low throughput due to high number of technical replicates Experienced staff needed for visual evaluation of beating 2D cell system Long assay duration
hiPS Test (this study)	Human	hiPSCs	2D	Cell viability Beating area Beating frequency mRNA expression	No FBS No ethical concerns using hiPSCs Defined percentage of cardiomyocytes Low number of replicates No human-based evaluation	Low throughput 2D cell system

EST—Embryonic stem cell test, ECVAM—European Centre for the Validation of Alternative Methods, FBS—Fetal bovine serum, hESC—human embryonic stem cell, iPST—Induced pluripotent stem cells testing, hiPSC—Human induced pluripotent stem cell, EB—Embryoid body, hiPS Test—Human induced pluripotent stem cell test.

**Table 5:** Antibodies and isotype controls used for flow cytometry analysis of human induced pluripotent stem cells (hiPSCs).

Conjugated Fluorochrome	Antibody	Isotype Control
PE	Mouse anti-human NANOG	Mouse IgG1, κ
PerCP-Cy™ 5.5	Mouse anti-OCT3/4	Mouse IgG1, κ
Alexa Fluor^®^ 647	Mouse anti-SOX2	Mouse IgG2a, κ
FITC	Mouse anti-SSEA-4 (BD, Franklin Lakes, NJ, USA, #560126)	Mouse IgG3, κ (BD, Franklin Lakes, NJ, USA, #555578)

## Data Availability

Not applicable.

## Supplementary Materials

The following supporting information can be downloaded at: https://www.mdpi.com/article/10.3390/ijms23063295/s1.

## Abbreviations

hiPSC(s)—human induced pluripotent stem cell(s); FBS—Fetal bovine serum; hiPS Test—human induced pluripotent stem cell test; REACH—Registration, Evaluation, Authorization and Restriction of Chemicals; ECHA—European Chemicals Agency; EFSA—European Food Safety Authority; EURL-ECVAM—European Union Reference Laboratory for Alternatives to Animal Testing; WEC—whole-embryo culture; MM—micromass test; EST—embryonic stem cell test; m/hESC(s)—mouse/human embryonic stem cell(s); 5-FU—5-Fluorouracil; PenG—Penicillin G; ESC(s)—embryonic stem cell(s); MCB—master cell bank; WCB—working cell bank; LN521—Laminin521; FVS—Fixable viability stain; p—passage; OCT3/4—octamer-binding transcription factor 4; SOX2—SRY-Box Transcription Factor 2; SSEA4—Stage-specific embryonic antigen-4; CHIR—CHIR99021; BMP—Bone morphogenetic protein; ISL1—ISL LIM Homeobox 1; IWP2—Inhibitor of Wnt Production-2; MG — Matrigel; MESP1—mesoderm posterior bHLH transcription factor 1; GATA4—GATA binding protein 4; TNNT2—troponin T2; cardiac type; ACTN2—Actinin Alpha 2; CANX—Calnexin; cTnT—cardiac troponin T; MEA—microelectrode arrays; EBs—embryoid bodies; FdUMP—fluorodeoxyuridine monophosphate; dTMP—deoxythymidine monophosphate; BMC—benchmark concentration; IC_50_—half-maximal inhibitory concentration of viability; ID_50_—half-maximal inhibitory concentration of differentiation; BMR—benchmark response; BMCL—benchmark concentration lower limit; BMCU—benchmark concentration upper limit; REP—Relative Embryotoxic Potency; TBX5—T-Box Transcription Factor 5; MEF2c—Myocyte Enhancer Factor 2C; HAND1—Heart And Neural Crest Derivatives Expressed 1; CMYA1—Cardiomyopathy associated 1; ROI—region of interest; HSL—hue; saturation; lightness; KO-DMEM—KnockOut^TM^ DMEM; RT—Room temperature; rh—recombinant human; CTB—CellTiter-Blue^®^.

## References

[1] Miller B.F., Keane C., O’Toole M.T. (2003). Encyclopedia & Dictionary of Medicine, Nursing & Allied Health.

[2] Nicoll R. (2018). Environmental Contaminants and Congenital Heart Defects: A Re-Evaluation of the Evidence. Int. J. Environ. Res. Public Health.

[3] MacDorman M.F., Gregory E.C.W. (2015). Fetal and Perinatal Mortality: United States, 2013. Natl. Vital Stat. Rep..

[4] DeSesso J.M. (2017). Future of Developmental Toxicity Testing. Curr. Opin. Toxicol..

[5] Weinhold B. (2009). Environmental Factors in Birth Defects: What We Need to Know. Environ. Health Perspect..

[6] (2010). EU directive 2010/63/EU Directive 2010/63/EU of the European Parliament and of the Council of 22 September 2010 on the Protection of Animals Used for Scientific Purposes. Off. J. Eur. Union.

[7] ECHA Understanding REACH. https://echa.europa.eu/regulations/reach/understanding-reach.

[8] Organisation for Economic Cooperation and Development OECD Guidelines for the Testing of Chemicals, Section 4. Health Effects. https://www.oecd-ilibrary.org/environment/oecd-guidelines-for-the-testing-of-chemicals-section-4-health-effects_20745788.

[9] USEPA (1991). Guidelines for Developmental Toxicity Risk Assessment.

[10] Pistollato F., Madia F., Corvi R., Munn S., Grignard E., Paini A., Worth A., Bal-Price A., Prieto P., Casati S. (2021). Current EU Regulatory Requirements for the Assessment of Chemicals and Cosmetic Products: Challenges and Opportunities for Introducing New Approach Methodologies. Arch. Toxicol..

[11] Rovida C., Hartung T. (2009). Re-Evaluation of Animal Numbers and Costs for in Vivo Tests to Accomplish REACH Legislation Requirements for Chemicals—A Report by the Transatlantic Think Tank for Toxicology (t(4)). ALTEX.

[12] Russell W.M.S., Burch R.L. (1959). The Principles of Humane Experimental Technique.

[13] Genschow E., Spielmann H., Scholz G., Seiler A., Brown N., Piersma A., Brady M., Clemann N., Huuskonen H., Paillard F. (2002). The ECVAM International Validation Study on in Vitro Embryotoxicity Tests: Results of the Definitive Phase and Evaluation of Prediction Models. ATLA Altern. Lab. Anim..

[14] Spielmann H., Seiler A., Bremer S., Hareng L., Hartung T., Ahr H., Faustman E., Haas U., Moffat G.J., Nau H. (2006). The Practical Application of Three Validated in Vitro Embryotoxicity Tests. ATLA Altern. Lab. Anim..

[15] National Research Council, National Research Council (2004). National Research Council Intentional Human Dosing Studies for EPA Regulatory Purposes: Scientific and Ethical Issues.

[16] Bailey J., Thew M., Balls M. (2015). Predicting Human Drug Toxicity and Safety via Animal Tests. Altern. Lab. Anim..

[17] Attarwala H. (2010). TGN1412: From Discovery to Disaster. J. Young Pharm..

[18] Leist M., Hartung T. (2013). Inflammatory Findings on Species Extrapolations: Humans Are Definitely No 70-Kg Mice. Arch. Toxicol..

[19] Seok J., Warren H.S., Cuenca A.G., Mindrinos M.N., Baker H.V., Xu W., Richards D.R., McDonald-Smith G.P., Gao H., Hennessy L. (2013). Genomic Responses in Mouse Models Poorly Mimic Human Inflammatory Diseases. Proc. Natl. Acad. Sci. USA.

[20] Shanks N., Greek R., Greek J. (2009). Are Animal Models Predictive for Humans?. Philos. Ethics Humanit. Med..

[21] Irie N., Kuratani S. (2011). Comparative Transcriptome Analysis Reveals Vertebrate Phylotypic Period during Organogenesis. Nat. Commun..

[22] Rayon T., Stamataki D., Perez-Carrasco R., Garcia-Perez L., Barrington C., Melchionda M., Exelby K., Lazaro J., Tybulewicz V.L.J., Fisher E.M.C. (2020). Species-Specific Pace of Development Is Associated with Differences in Protein Stability. Science.

[23] Xue L., Cai J.-Y., Ma J., Huang Z., Guo M.-X., Fu L.-Z., Shi Y.-B., Li W.-X. (2013). Global Expression Profiling Reveals Genetic Programs Underlying the Developmental Divergence between Mouse and Human Embryogenesis. BMC Genom..

[24] Uosaki H., Taguchi Y. (2016). Comparative Gene Expression Analysis of Mouse and Human Cardiac Maturation. Genom. Proteom. Bioinform..

[25] Olson H., Betton G., Robinson D., Thomas K., Monro A., Kolaja G., Lilly P., Sanders J., Sipes G., Bracken W. (2000). Concordance of the Toxicity of Pharmaceuticals in Humans and in Animals. Regul. Toxicol. Pharmacol..

[26] Takahashi K., Tanabe K., Ohnuki M., Narita M., Ichisaka T., Tomoda K., Yamanaka S. (2007). Induction of Pluripotent Stem Cells from Adult Human Fibroblasts by Defined Factors. Cell.

[27] Fritsche E., Haarmann-Stemmann T., Kapr J., Galanjuk S., Hartmann J., Mertens P.R., Kämpfer A.A.M., Schins R.P.F., Tigges J., Koch K. (2021). Stem Cells for Next Level Toxicity Testing in the 21st Century. Small.

[28] Zink D., Chuah J.K.C., Ying J.Y. (2020). Assessing Toxicity with Human Cell-Based In Vitro Methods. Trends Mol. Med..

[29] Matsui T., Miyamoto N., Saito F., Shinozawa T. (2020). Molecular Profiling of Human Induced Pluripotent Stem Cell-Derived Cells and Their Application for Drug Safety Study. Curr. Pharm. Biotechnol..

[30] Smith A.S.T., Macadangdang J., Leung W., Laflamme M.A., Kim D.-H. (2017). Human IPSC-Derived Cardiomyocytes and Tissue Engineering Strategies for Disease Modeling and Drug Screening. Biotechnol. Adv..

[31] Stacey G.N., Crook J.M., Hei D., Ludwig T. (2013). Banking Human Induced Pluripotent Stem Cells: Lessons Learned from Embryonic Stem Cells?. Cell Stem Cell.

[32] Tigges J., Bielec K., Brockerhoff G., Hildebrandt B., Hübenthal U., Kapr J., Koch K., Teichweyde N., Wieczorek D., Rossi A. (2021). Academic Application of Good Cell Culture Practice for Induced Pluripotent Stem Cells. ALTEX.

[33] Frank S., Zhang M., Schöler H.R., Greber B. (2012). Small Molecule-Assisted, Line-Independent Maintenance of Human Pluripotent Stem Cells in Defined Conditions. PLoS ONE.

[34] Wakui T. (2017). Method for Evaluation of Human Induced Pluripotent Stem Cell Quality Using Image Analysis Based on the Biological Morphology of Cells. J. Med. Imaging.

[35] Ye H., Wang Q. (2018). Efficient Generation of Non-Integration and Feeder-Free Induced Pluripotent Stem Cells from Human Peripheral Blood Cells by Sendai Virus. Cell. Physiol. Biochem..

[36] Emre N., Vidal J.G., Elia J., O’Connor E.D., Paramban R.I., Hefferan M.P., Navarro R., Goldberg D.S., Varki N.M., Marsala M. (2010). The ROCK Inhibitor Y-27632 Improves Recovery of Human Embryonic Stem Cells after Fluorescence-Activated Cell Sorting with Multiple Cell Surface Markers. PLoS ONE.

[37] Sullivan S., Stacey G.N., Akazawa C., Aoyama N., Baptista R., Bedford P., Bennaceur Griscelli A., Chandra A., Elwood N., Girard M. (2018). Quality Control Guidelines for Clinical-Grade Human Induced Pluripotent Stem Cell Lines. Regen. Med..

[38] Pamies D., Bal-Price A., Simeonov A., Tagle D., Allen D., Gerhold D., Yin D., Pistollato F., Inutsuka T., Sullivan K. (2017). Good Cell Culture Practice for Stem Cells and Stem-Cell-Derived Models. ALTEX Altern. Anim. Exp..

[39] Lian X., Zhang J., Azarin S.M., Zhu K., Hazeltine L.B., Bao X., Hsiao C., Kamp T.J., Palecek S.P. (2013). Directed Cardiomyocyte Differentiation from Human Pluripotent Stem Cells by Modulating Wnt/β-Catenin Signaling under Fully Defined Conditions. Nat. Protoc..

[40] Bai Q., Ramirez J.M., Becker F., Pantesco V., Lavabre-Bertrand T., Hovatta O., Lemaître J.M., Pellestor F., De Vos J. (2015). Temporal Analysis of Genome Alterations Induced by Single-Cell Passaging in Human Embryonic Stem Cells. Stem Cells Dev..

[41] Mayshar Y., Ben-David U., Lavon N., Biancotti J.C., Yakir B., Clark A.T., Plath K., Lowry W.E., Benvenisty N. (2010). Identification and Classification of Chromosomal Aberrations in Human Induced Pluripotent Stem Cells. Cell Stem Cell.

[42] Ben-David U., Benvenisty N., Mayshar Y. (2010). Genetic Instability in Human Induced Pluripotent Stem Cells: Classification of Causes and Possible Safeguards. Cell Cycle.

[43] Laurent L.C., Ulitsky I., Slavin I., Tran H., Schork A., Morey R., Lynch C., Harness J.V., Lee S., Barrero M.J. (2011). Dynamic Changes in the Copy Number of Pluripotency and Cell Proliferation Genes in Human ESCs and IPSCs during Reprogramming and Time in Culture. Cell Stem Cell.

[44] WHO (2013). Annex 3 Recommendations for the Evaluation of Animal Cell Cultures as Substrates for the Manufacture of Biological Medicinal Products and for the Characterization of Cell Banks Replacement of Annex 1 of WHO Technical Report Series, No. 878.

[45] Assou S., Bouckenheimer J., De Vos J. (2018). Concise Review: Assessing the Genome Integrity of Human Induced Pluripotent Stem Cells: What Quality Control Metrics?. Stem Cells.

[46] Zhang M., Schulte J.S., Heinick A., Piccini I., Rao J., Quaranta R., Zeuschner D., Malan D., Kim K.-P., Röpke A. (2015). Universal Cardiac Induction of Human Pluripotent Stem Cells in Two and Three-Dimensional Formats: Implications for In Vitro Maturation. Stem Cells.

[47] Naito A.T., Shiojima I., Akazawa H., Hidaka K., Morisaki T., Kikuchi A., Komuro I. (2006). Developmental Stage-Specific Biphasic Roles of Wnt/β-Catenin Signaling in Cardiomyogenesis and Hematopoiesis. Proc. Natl. Acad. Sci. USA.

[48] Tian Y., Cohen E.D., Morrisey E.E. (2010). The Importance of Wnt Signaling in Cardiovascular Development. Pediatr. Cardiol..

[49] Garitaonandia I., Amir H., Boscolo F.S., Wambua G.K., Schultheisz H.L., Sabatini K., Morey R., Waltz S., Wang Y.C., Tran H. (2015). Increased Risk of Genetic and Epigenetic Instability in Human Embryonic Stem Cells Associated with Specific Culture Conditions. PLoS ONE.

[50] Shi G., Jin Y. (2010). Role of Oct4 in Maintaining and Regaining Stem Cell Pluripotency. Stem Cell Res. Ther..

[51] Saga Y., Kitajima S., Miyagawa-Tomita S., Takagi A., Kitajima S., Miyazaki J., Inoue T. (1999). MesP1 Is Expressed in the Heart Precursor Cells and Required for the Formation of a Single Heart Tube. Trends Cardiovasc. Med..

[52] Bondue A., Blanpain C. (2010). Mesp1: A Key Regulator of Cardiovascular Lineage Commitment. Circ. Res..

[53] Lindsley R.C., Gill J.G., Murphy T.L., Langer E.M., Cai M., Mashayekhi M., Wang W., Niwa N., Nerbonne J.M., Kyba M. (2008). Mesp1 Coordinately Regulates Cardiovascular Fate Restriction and Epithelial-Mesenchymal Transition in Differentiating ESCs. Cell Stem Cell.

[54] Ueno S., Weidinger G., Osugi T., Kohn A.D., Golob J.L., Pabon L., Reinecke H., Moon R.T., Murry C.E. (2007). Biphasic Role for Wnt/β-Catenin Signaling in Cardiac Specification in Zebrafish and Embryonic Stem Cells. Proc. Natl. Acad. Sci. USA.

[55] Cohen E.D., Tian Y., Morrisey E.E. (2008). Wnt Signaling: An Essential Regulator of Cardiovascular Differentiation, Morphogenesis and Progenitor Self-Renewal. Development.

[56] Cai C.-L.L., Liang X., Shi Y., Chu P.-H.H., Pfaff S.L., Chen J., Evans S. (2003). Isl1 Identifies a Cardiac Progenitor Population That Proliferates Prior to Differentiation and Contributes a Majority of Cells to the Heart. Dev. Cell.

[57] Brade T., Pane L.S., Moretti A., Chien K.R., Laugwitz K.-L. (2013). Embryonic Heart Progenitors and Cardiogenesis. Cold Spring Harb. Perspect. Med..

[58] England J., Pang K.L., Parnall M., Haig M.I., Loughna S. (2016). Cardiac Troponin T Is Necessary for Normal Development in the Embryonic Chick Heart. J. Anat..

[59] Tiso N., Majetti M., Stanchi F., Rampazzo A., Zimbello R., Nava A., Danieli G.A. (1999). Fine Mapping and Genomic Structure of ACTN2, the Human Gene Coding for the Sarcomeric Isoform of α-Actinin-2, Expressed in Skeletal and Cardiac Muscle. Biochem. Biophys. Res. Commun..

[60] Holmgren G., Ghosheh N., Zeng X., Bogestål Y., Sartipy P., Synnergren J. (2015). Identification of Stable Reference Genes in Differentiating Human Pluripotent Stem Cells. Physiol. Genomics.

[61] Holmgren G., Zeng X., Synnergren J. Selection of Robust Reference Genes for Normalization of Quantitative RT-PCR Data from Differentiating Human Pluripotent Stem Cells. Proceedings of the 7th ACM International Conference on Bioinformatics and Computational Biology, BICOB.

[62] Zhu H., Scharnhorst K.S., Stieg A.Z., Gimzewski J.K., Minami I., Nakatsuji N., Nakano H., Nakano A. (2017). Two Dimensional Electrophysiological Characterization of Human Pluripotent Stem Cell-Derived Cardiomyocyte System. Sci. Rep..

[63] Seki T., Yuasa S., Kusumoto D., Kunitomi A., Saito Y., Tohyama S., Yae K., Kishino Y., Okada M., Hashimoto H. (2014). Generation and Characterization of Functional Cardiomyocytes Derived from Human T Cell-Derived Induced Pluripotent Stem Cells. PLoS ONE.

[64] Balafkan N., Mostafavi S., Schubert M., Siller R., Liang K.X., Sullivan G., Bindoff L.A. (2020). A Method for Differentiating Human Induced Pluripotent Stem Cells toward Functional Cardiomyocytes in 96-Well Microplates. Sci. Rep..

[65] Lauschke K., Rosenmai A.K., Meiser I., Neubauer J.C., Schmidt K., Rasmussen M.A., Holst B., Taxvig C., Emnéus J.K., Vinggaard A.M. (2020). A Novel Human Pluripotent Stem Cell-Based Assay to Predict Developmental Toxicity. Arch. Toxicol..

[66] Snir M., Kehat I., Gepstein A., Coleman R., Itskovitz-Eldor J., Livne E., Gepstein L. (2003). Assessment of the Ultrastructural and Proliferative Properties of Human Embryonic Stem Cell-Derived Cardiomyocytes. Am. J. Physiol.-Heart Circ. Physiol..

[67] Ohashi F., Miyagawa S., Yasuda S., Miura T., Kuroda T., Itoh M., Kawaji H., Ito E., Yoshida S., Saito A. (2019). CXCL4/PF4 Is a Predictive Biomarker of Cardiac Differentiation Potential of Human Induced Pluripotent Stem Cells. Sci. Rep..

[68] Ng A.H.M., Khoshakhlagh P., Rojo Arias J.E., Pasquini G., Wang K., Swiersy A., Shipman S.L., Appleton E., Kiaee K., Kohman R.E. (2021). A Comprehensive Library of Human Transcription Factors for Cell Fate Engineering. Nat. Biotechnol..

[69] Burridge P.W., Matsa E., Shukla P., Lin Z.C., Churko J.M., Ebert A.D., Lan F., Diecke S., Huber B., Mordwinkin N.M. (2014). Chemically Defned Generation of Human Cardiomyocytes. Nat. Methods.

[70] Fleischer S., Jahnke H.G., Fritsche E., Girard M., Robitzki A.A. (2019). Comprehensive Human Stem Cell Differentiation in a 2D and 3D Mode to Cardiomyocytes for Long-Term Cultivation and Multiparametric Monitoring on a Multimodal Microelectrode Array Setup. Biosens. Bioelectron..

[71] Zwartsen A., de Korte T., Nacken P., de Lange D.W., Westerink R.H.S., Hondebrink L. (2019). Cardiotoxicity Screening of Illicit Drugs and New Psychoactive Substances (NPS) in Human IPSC-Derived Cardiomyocytes Using Microelectrode Array (MEA) Recordings. J. Mol. Cell. Cardiol..

[72] Aikawa N. (2020). A Novel Screening Test to Predict the Developmental Toxicity of Drugs Using Human Induced Pluripotent Stem Cells. J. Toxicol. Sci..

[73] Walker L.M., Sparks N.R.L., Puig-Sanvicens V., Rodrigues B., Zur Nieden N.I. (2021). An Evaluation of Human Induced Pluripotent Stem Cells to Test for Cardiac Developmental Toxicity. Int. J. Mol. Sci..

[74] Genschow E., Spielmann H., Scholz G., Pohl I., Seiler A., Clemann N., Bremer S., Becker K. (2004). Validation of the Embryonic Stem Cell Test in the International ECVAM Validation Study on Three In Vitro Embryotoxicity Tests. Altern. Lab. Anim..

[75] Seiler A.E.M., Spielmann H. (2011). The Validated Embryonic Stem Cell Test to Predict Embryotoxicity in Vitro. Nat. Protoc..

[76] Zhang N., Yin Y., Xu S.-J., Chen W.-S. (2008). 5-Fluorouracil: Mechanisms of Resistance and Reversal Strategies. Molecules.

[77] Shuey D.L., Lau C., Logsdon T.R., Zucker R.M., Elstein K.H., Narotsky M.G., Setzer R.W., Kavlock R.J., Rogers J.M. (1994). Biologically Based Dose-Response Modeling in Developmental Toxicology: Biochemical and Cellular Sequelae of 5-Fluorouracil Exposure in the Developing Rat. Toxicol. Appl. Pharmacol..

[78] Lau C., Mole M.L., Copeland M.F., Rogers J.M., Kavlock R.J., Shuey D.L., Cameron A.M., Ellis D.H., Logsdon T.R., Merriman J. (2001). Toward a Biologically Based Dose-Response Model for Developmental Toxicity of 5-Fluorouracil in the Rat: Acquisition of Experimental Data. Toxicol. Sci..

[79] Schaefer C., Spielmann H., Vetter K., Weber-Schöndorfer C. (2006). Spezielle Arzneimitteltherapie in Der Schwangerschaft. Arzneiverordnung in Schwangerschaft und Stillzeit.

[80] Nathan L., Bawdon R.E., Sidawi J.E., Stettler R.W., McIntire D.M., Wendel G.D. (1993). Penicillin Levels Following the Administration of Benzathine Penicillin G in Pregnancy. Obstet. Gynecol..

[81] Weeks J.W., Myers S.R., Lasher L., Goldsmith J., Watkins C., Gall S.A. (1997). Persistence of Penicillin G Benzathine in Pregnant Group B Streptococcus Carriers. Obstet. Gynecol..

[82] Crofton K.M., Mundy W.R., Lein P.J., Bal-Price A., Coecke S., Seiler A.E.M., Knaut H., Buzanska L., Goldberg A. (2011). Developmental Neurotoxicity Testing: Recommendations for Developing Alternative Methods for the Screening and Prioritization of Chemicals. ALTEX.

[83] Adler S., Pellizzer C., Hareng L., Hartung T., Bremer S. (2008). First Steps in Establishing a Developmental Toxicity Test Method Based on Human Embryonic Stem Cells. Toxicol. Vitr..

[84] Berrouet C., Dorilas N., Rejniak K.A., Tuncer N. (2020). Comparison of Drug Inhibitory Effects ($$\hbox {IC}_{50}$$ IC 50 ) in Monolayer and Spheroid Cultures. Bull. Math. Biol..

[85] Hardy A., Benford D., Halldorsson T., Jeger M.J., Knutsen K.H., More S., Mortensen A., Naegeli H., Noteborn H., Ockleford C. (2017). Update: Use of the Benchmark Dose Approach in Risk Assessment. EFSA J..

[86] Krebs A., Nyffeler J., Karreman C., Schmidt B.Z., Kappenberg F., Mellert J., Pallocca G., Pastor M., Rahnenführer J., Leist M. (2020). Determination of Benchmark Concentrations and Their Statistical Uncertainty for Cytotoxicity Test Data and Functional In Vitro Assays. ALTEX.

[87] Masjosthusmann S., Blum J., Bartmann K., Dolde X., Holzer A., Stürzl L., Keßel E.H., Förster N., Dönmez A., Klose J. (2020). Establishment of an a Priori Protocol for the Implementation and Interpretation of an In-vitro Testing Battery for the Assessment of Developmental Neurotoxicity. EFSA Support..

[88] Zhao Q., Sun Q., Zhou L., Liu K., Jiao K. (2019). Complex Regulation of Mitochondrial Function During Cardiac Development. J. Am. Heart Assoc..

[89] Yamada S., Yamazaki D., Kanda Y. (2018). 5-Fluorouracil Inhibits Neural Differentiation via Mfn1/2 Reduction in Human Induced Pluripotent Stem Cells. J. Toxicol. Sci..

[90] Pellizzer C., Adler S., Corvi R., Hartung T., Bremer S. (2004). Monitoring of Teratogenic Effects in Vitro by Analysing a Selected Gene Expression Pattern. Toxicol. Vitr..

[91] Peters A.K., Steemans M., Mesens N., Hansen E., Verheyen G.R., Spanhaak S., Coussement W., Vanparys P. (2007). A Higher Throughput Method to the Embryonic Stem Cell Test (EST), to Detect Embryotoxicity in Early Development. AATEX.

[92] Peters A.K., Wouwer G., Van de Weyn B., Verheyen G.R., Vanparys P., Van Gompel J. (2008). Automated Analysis of Contractility in the Embryonic Stem Cell Test, a Novel Approach to Assess Embryotoxicity. Toxicol. Vitr..

[93] van Dartel D.A.M., Pennings J.L.A., de la Fonteyne L.J.J., van Herwijnen M.H., van Delft J.H., van Schooten F.J., Piersma A.H. (2010). Monitoring Developmental Toxicity in the Embryonic Stem Cell Test Using Differential Gene Expression of Differentiation-Related Genes. Toxicol. Sci..

[94] Suzuki N., Ando S., Yamashita N., Horie N., Saito K. (2011). Evaluation of Novel High-Throughput Embryonic Stem Cell Tests with New Molecular Markers for Screening Embryotoxic Chemicals in Vitro. Toxicol. Sci..

[95] Witt G., Keminer O., Leu J., Tandon R., Meiser I., Willing A., Winschel I., Abt J.-C., Brändl B., Sébastien I. (2021). An Automated and High-Throughput-Screening Compatible Pluripotent Stem Cell-Based Test Platform for Developmental and Reproductive Toxicity Assessment of Small Molecule Compounds. Cell Biol. Toxicol..

[96] Gstraunthaler G., Lindl T., van der Valk J. (2013). A Plea to Reduce or Replace Fetal Bovine Serum in Cell Culture Media. Cytotechnology.

[97] Lo B., Parham L. (2009). Ethical Issues in Stem Cell Research. Endocr. Rev..

[98] Bobbert M. (2006). Ethical Questions Concerning Research on Human Embryos, Embryonic Stem Cells and Chimeras. Biotechnol. J..

[99] Steimle J.D., Moskowitz I.P. (2017). TBX5: A Key Regulator of Heart Development. Curr. Top. Dev. Biol..

[100] Bradski G. The OpenCV Library. https://www.drdobbs.com/open-source/the-opencv-library/184404319.

[101] Dach K., Bendt F., Huebenthal U., Giersiefer S., Lein P.J., Heuer H., Fritsche E. (2017). BDE-99 Impairs Differentiation of Human and Mouse NPCs into the Oligodendroglial Lineage by Species-Specific Modes of Action. Sci. Rep..

[102] Ritz C., Baty F., Streibig J.C., Gerhard D. (2015). Dose-Response Analysis Using R. PLoS ONE.

