# Supplementary material for: The Human Induced Pluripotent Stem Cell Test as an Alternative Method for Embryotoxicity Testing
Source: Int J Mol Sci. 2022 Mar 18;23(6):3295. doi: 10.3390/ijms23063295 (PMC8950674; doi:10.3390/ijms23063295)
Supplement: Supplementary file 1 [file ijms-23-03295-s001.zip › ijms-1610636-suppl/Supplementary material_proofread.pdf]

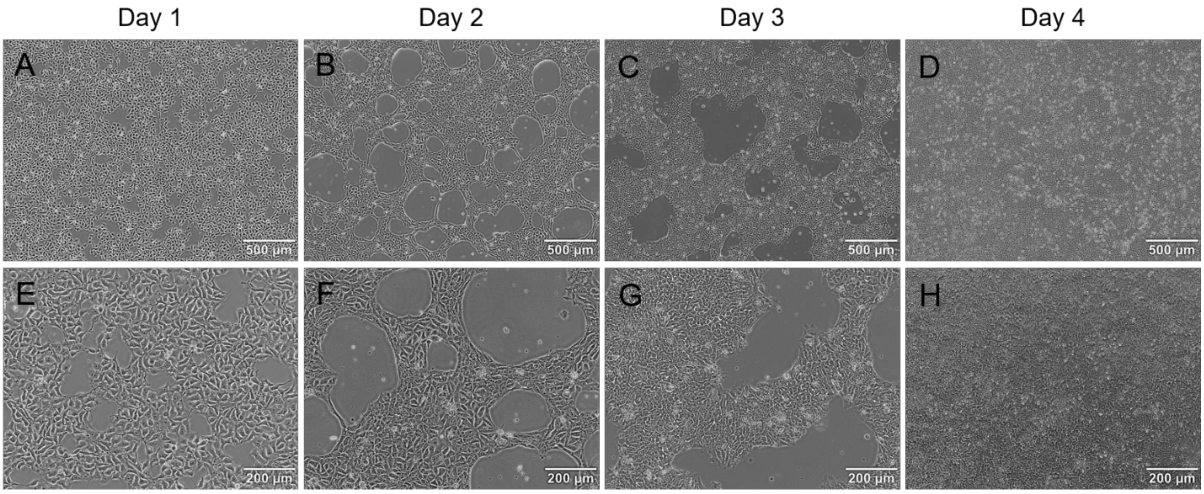

Figure S1: Exemplary phase-contrast images of the human induced pluripotent stem cell (hiPSC) line iPS11 as a function of culture time. Human iPSCs were seeded with a density of $2x10^5$ cells/ Laminin521 (LN521)-coated 6-well for four days as described in“Cell Passaging”. Cells were in passage 6 after thawing. Images of the same well were taken with an Olympus CKX53SF (Tokyo, Japan) with an integrated camera SC50. **A-D:** Magnification: 40x **E-H:** Magnification: 100x .

Video S1: Exemplary video recordings of beating cardiomyocytes derived from human induced pluripotent stem cells (hiPSCs) on day 10 of hiPSC passages 4, 6, 8, 10, and 20. Cells were differentiated according to the protocol in Figure 3A. Videos were recorded on day 10 of the protocol with a binocular (Leica, Wetzlar, Germany), magnification 1x. **A-E:** Cardiomyocytes differentiated from the hiPSC line iPS11 in passages 4, 6, 8, 10, and 20, respectively.

Video S2: Exemplary video recordings of differentiated cardiomyocytes generated in the range finding experiment for CHIR99021 (CHIR) and bone morphogenetic protein 4 (BMP4) shown in Figure 3C. Concentrations of CHIR and BMP4 were assessed in a grid ranging from 1.5 to 2 µM CHIR and 0.5 to 1 ng/mL BMP4 in increments of 0.25 µM and ng/mL, respectively. Concentrations were tested in triplicates seeding $2.75x10^5$ cells/Matrigel (MG)-coated 48-well following the protocol shown in Figure 3A. Videos were taken on day 10 using a binocular Leica DMS1000 B (Wetzlar, Germany) with an integrated heating plate set to 37°C. Magnification: 1x.

Video S3: Exemplary video recordings of differentiated cardiomyocytes on days 8, 9, and 10. Human induced pluripotent stem cells (hiPSCs) were differentiated according to the protocol shown in Figure 3A. Videos were taken on days 8, 9, and 10, respectively, using a binocular Leica DMS1000 B (Wetzlar, Germany) with an integrated heating plate set to 37°C. Magnification: 1x. **A:** Beating cardiomyocytes on day 8 with multiple starting points. **B:** On day 9 the beating of cardiomyocytes is more synchronized compared to day 8. **C:** Cardiomyocytes beating in a synchronized wave over the entire well on day 10. The beating is more pronounced compared to days 8 and 9.

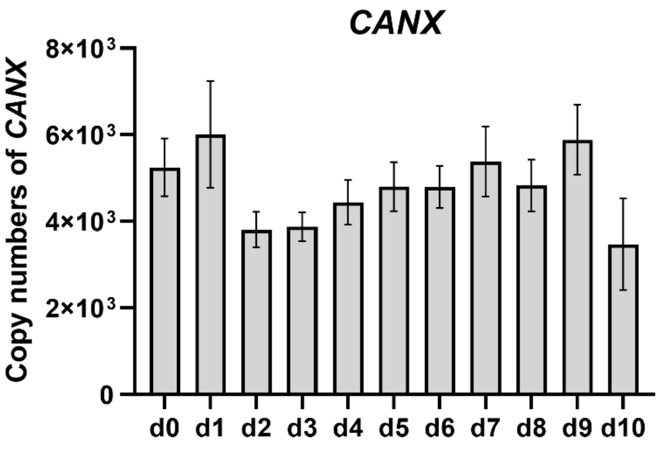

Figure S2: Expression of the reference gene CANX in human induced pluripotent stem cell (hiPSC)-derived cardiomyocytes within a time course of 10 days. Triplicates of $2.75x10^5$ hiPSCs/well were seeded onto Matrigel-coated 48-well plates and differentiated according to the protocol shown in Figure 3A. Each day a triplicate was collected and pooled for RT-qPCR analysis of reference gene CANX. For the time point 'd0', 1-2 million hiPSCs were collected during the splitting procedure of the respective experiment. N = 4, ± SEM, p = 0.05, significant compared to each other.

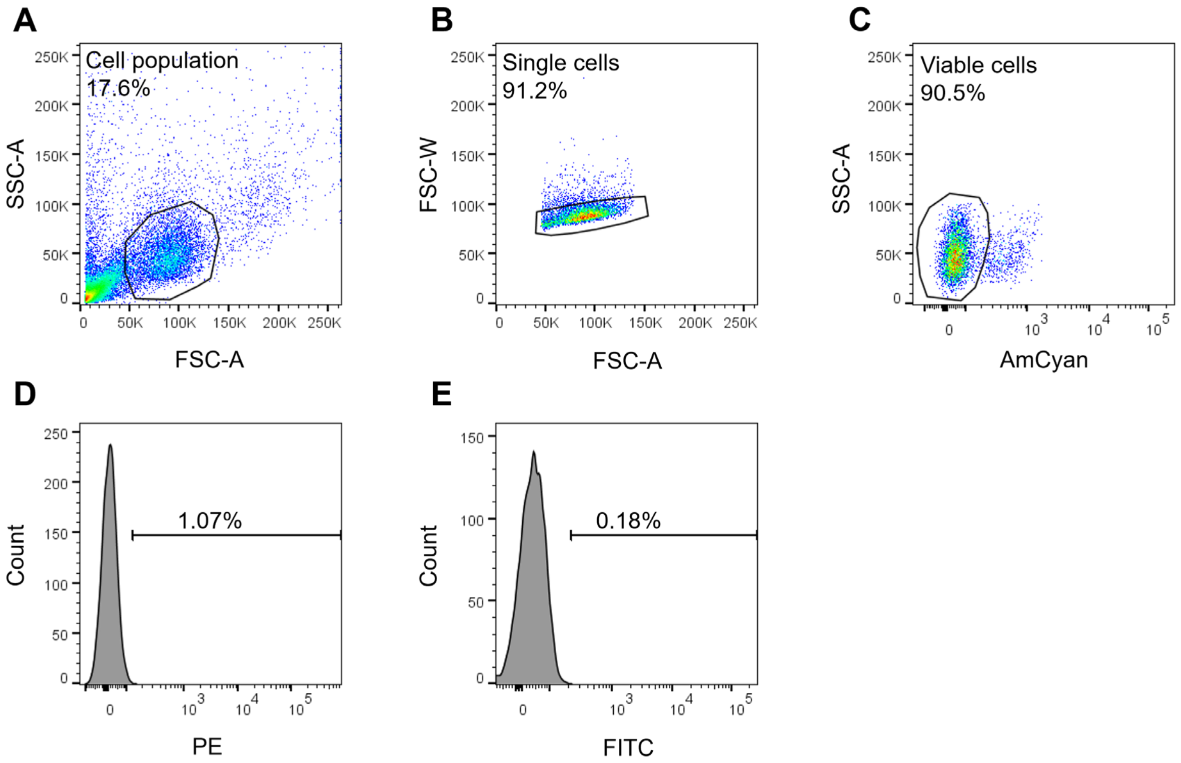

Figure S3: Exemplary gating strategy for the flow cytometry analyses of human induced pluripotent stem cell (hiPSC)-derived cardiomyocytes on day 10 analyzing cardiomyocyte-specific markers cardiac muscle Troponin T (cTnT)-PE and α-Actinin2-FITC plus Fixable Viability Stain (FVS) 510 as a live/dead discriminator. Human iPSCs were differentiated into cardiomyocytes according to the protocol in Figure 3A. The acquisition was performed using a BD FACSCanto™ II system operated with the BD FACSDiva™ software. Further analysis was conducted with FlowJo. **A:** Gating strategy for the relevant cell population **B:** Gating strategy to ensure analyses of single cells **C:** Gating strategy to discriminate between live and dead cells **D-E:** Isotype controls for the respective antibodies used. The gate was

set to a maximum of 0.49% positive cells in the unstained control, every signal above was counted as a positive signal.

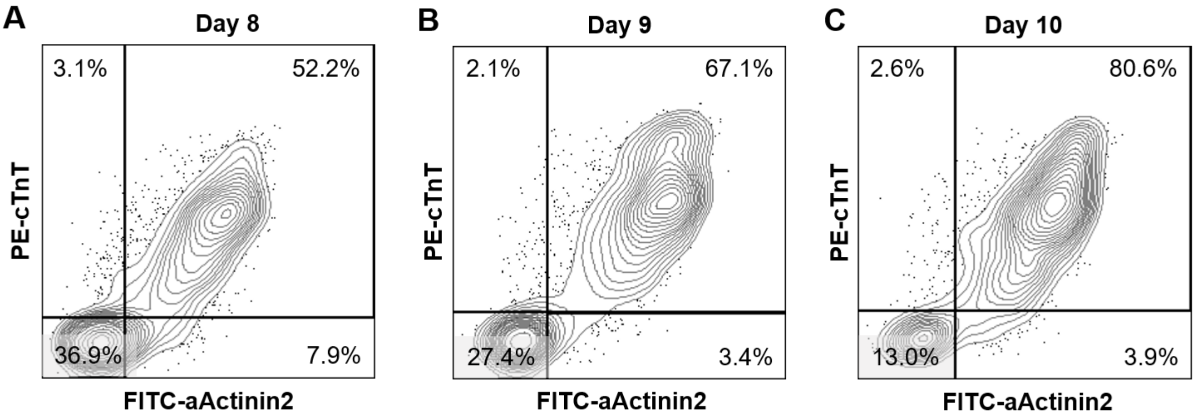

Figure S4: Exemplary flow cytometry analysis of human induced pluripotent stem cell (hiPSC)-derived cardiomyocytes analyzing cardiac-specific markers cardiac muscle Troponin T (cTnT)-PE and α-Actinin2-FITC on days 8, 9, and 10. Human iPSCs were differentiated into cardiomyocytes according to the protocol in Figure 3A. The acquisition was performed using a BD (Franklin Lakes, NJ, USA) FACSCanto^{TM} II system operated with the BD FACSDiva™ software. Further analysis was conducted with FlowJo. **A-C:** Contour plots of cells positive for cTnT and α-Actinin2 on days 8, 9, and 10, respectively.

Table S1: Composition of the FTDA medium for human induced plurpotent stem cell (hiPSC) culture adapted from Frank et al. [33].

**Component**	**Company, City, Country, Order number**	**Stock**	**Final concentration**
DMEM/F12 -L-glutamine	Thermo Fisher Scientific, Waltham, MA, USA, #21331020	-	-
Lipid-Mix	Thermo Fisher Scientific, Waltham, MA, USA, #11905031	100 %	1 %
Human serum albumin	Biological Industries, Beit-Haemek, Israel, #05-720-1B	10 %	0,1 %
L-Glutamine	Thermo Fisher Scientific, Waltham, MA, USA, #25030024	200 mM	2 mM
Penicillin<br>Streptomycin	PAN-Biotech GmbH, Aidenbach, Germany, #P06-07050	10,000 U/mL<br>10 mg/mL	100 U/mL<br>0,1 mg/mL
Animal-Free rhFGF-basic 154 a.a.	PeproTech, Cranbury, NJ, USA, #AF-100-18B	25 µg/mL	50 ng/mL

ITS Premix Universal Culture Supplement	Corning, Corning, NY, USA, #354351	Insulin 5 µg/mL<br>Transferrin 5 µg/mL<br>Selenious Acid 5 ng/mL	Insulin 5 ng/mL<br>Transferrin 5 ng/mL<br>Selenious Acid 5 pg/mL
rhActivin A	Stemcell Technologies, Vancouver, British Columbia, Canada #78001	10 µg/mL	4 ng/mL
TGFβ1	Thermo Fisher Scientific, Waltham, MA, USA, #34834882 and #PHG9204	1 µg/mL	0,2 ng/mL
Dorsomorphin	Tocris, Bristol, UK, #3093	500 µM	50 nM

DMEM/F12 - Dulbecco's Modified Eagle Medium/Nutrient Mixture F-12; rh – recombinant human; FGF - Fibroblast growth factor; TGFβ – Transforming growth factor β

Table S2: Composition of ITS medium for the induction of the cardiomyocyte differentiation on day 0 adapted from Zhang et al. [46].

Component	Company, City, Country, Order number	Stock	Final concentration
KnockOut™ DMEM	Thermo Fisher Scientific, Waltham, MA, USA, #10829018	-	-
L-Glutamine	Thermo Fisher Scientific, Waltham, MA, USA, #25030024	200 mM	2 mM
Penicillin<br>Streptomycin	PAN-Biotech GmbH, Aidenbach, Germany #P06-07050	10,000 U/ml<br>10 mg/ml	100 U/mL<br>0,1 mg/mL
ITS Premix Universal Culture Supplement	Corning, Corning, NY, USA, #354351	Insulin 5 µg/mL<br>Transferrin 5 µg/mL<br>Selenious Acid 5 ng/mL	Insulin 5 ng/mL<br>Transferrin 5 ng/mL<br>Selenious Acid 5 pg/mL

Y-27632	HelloBio, Bristol, UK, #HB2297	10 mM	10 µM
Animal-Free rhFGF-basic 154 a.a.	PeproTech, Cranbury, NJ, USA, #AF-100-18B	25 µg/mL	25 ng/mL
rhBMP4	R&D Systems, Minneapolis, MN, USA #314-BP	10 µg/mL	0,75 ng/mL
CHIR99021	Tocris, Bristol, UK, #4423	10 mM	1,75 µM

DMEM – Dulbecco's Modified Eagle Medium; Y-27632 – Rho-Associated Coil Kinase (ROCK) inhibitor; rh – recombinant human; FGF – Fibroblast growth factor, BMP4 – bone morphogenetic protein 4

Table S3: Composition of the TS medium for human induced pluripotent stem cell (hiPSC)-derived cardiomyocyte culture adapted from Zhang et al. [46].

Component	Company, City, Country, Order number	Stock	Final concentration
KnockOut™ DMEM	Thermo Fisher Scientific, Waltham, MA, USA #10829018	-	-
L-Glutamine	Thermo Fisher Scientific, Waltham, MA, USA #25030024	200 mM	2 mM

Penicillin<br>Streptomycin		PAN-Biotech GmbH, Aidenbach, Germany, #P06-07050	10,000 U/mL<br>10 mg/mL	100 U/mL<br>0,1 mg/mL
TS (x100)	hTransferrin	Merck, Darmstadt, Germany, #T8158	0,55 mg/mL	5,5 µg/mL
	Sodium selenite	Merck, Darmstadt, Germany, #S5261	0,67 µg/mL	6.7 ng/mL
2-Phospho-L-Ascorbic Acid		FUJIFILM Wako Pure Chemical Corporation, Osaka, Japan, #01312061	250 mM	250 µM

DMEM – Dulbecco's Modified Eagle Medium; h – human

Table S4: Primer sequences of forward (FW) and reverse (RV) primers used for RT-qPCR analysis.

Gene	Sequence (5‘-3‘)		Product length in bp
ACTN2	FW:	CTCAGACGCTCGTTAGCAT	150
	RV:	CAACATCAGAATCAGCTCAAGC	
CANX	FW:	GCTGGTTAGATGATGAGCCTGAG	139
	RV:	ACACCACATCCAGGAGCTGACT	
GATA4	FW:	TCCAAACCAGAAAACGGAAG	352
	RV:	AAGACCAGGCTGTTCCAAGA	
ISL1	FW:	CACAAGCGTCTCGGGATT	202
	RV:	AGTGGCAAGTCTTCCGACA	
MESP1	FW:	CTGCCTGAGGAGCCCAAGT	102
	RV:	GCAGTCTGCCAAGGAACCA	
POU5F1/ OCT4	FW:	GTTGGAGGGAAGGTGAAGTTC	103
	RV:	TGTGTCTATCTACTGTGTCCCA	
TNNT2	FW:	TTCACCAAAGATCTGCTCCTCGCT	166
	RV:	TTATTACTGGTGTGGAGTGGGTGTGG	

FW – forward; RV – reverse; bp – base pair; ACTN2 – Actinin Alpha 2; CANX – Calnexin; GATA4 – GATA binding protein 4; ISL1 – ISL LIM Homeobox 1; MESP1 – mesoderm posterior bHLH transcription factor 1; POU5F1/OCT4 – POU class 5 homeobox 1; TNNT2 – troponin T2, cardiac type
